# Developmental stage of transplanted neural progenitor cells influences anatomical and functional outcomes after spinal cord injury in mice

**DOI:** 10.1038/s42003-023-04893-0

**Published:** 2023-05-19

**Authors:** Miriam Aceves, Ashley Tucker, Joseph Chen, Katie Vo, Joshua Moses, Prakruthi Amar Kumar, Hannah Thomas, Diego Miranda, Gabrielle Dampf, Valerie Dietz, Matthew Chang, Aleena Lukose, Julius Jang, Sneha Nadella, Tucker Gillespie, Christian Trevino, Andrew Buxton, Anna L. Pritchard, Peyton Green, Dylan A. McCreedy, Jennifer N. Dulin

**Affiliations:** 1 Department of Biology, Texas A&M University, College Station, TX 77843 USA; 2 Texas A&M Institute for Neuroscience, Texas A&M University, College Station, TX 77843 USA; 3 Department of Biomedical Engineering, Texas A&M University, College Station, TX 77843 USA; 4 Ganado High School, Ganado, TX 77962 USA

**Keywords:** Spinal cord injury, Neural stem cells

## Abstract

Neural progenitor cell (NPC) transplantation is a promising therapeutic strategy for replacing lost neurons following spinal cord injury (SCI). However, how graft cellular composition influences regeneration and synaptogenesis of host axon populations, or recovery of motor and sensory functions after SCI, is poorly understood. We transplanted developmentally-restricted spinal cord NPCs, isolated from E11.5-E13.5 mouse embryos, into sites of adult mouse SCI and analyzed graft axon outgrowth, cellular composition, host axon regeneration, and behavior. Earlier-stage grafts exhibited greater axon outgrowth, enrichment for ventral spinal cord interneurons and Group-Z spinal interneurons, and enhanced host 5-HT^+^ axon regeneration. Later-stage grafts were enriched for late-born dorsal horn interneuronal subtypes and Group-N spinal interneurons, supported more extensive host CGRP^+^ axon ingrowth, and exacerbated thermal hypersensitivity. Locomotor function was not affected by any type of NPC graft. These findings showcase the role of spinal cord graft cellular composition in determining anatomical and functional outcomes following SCI.

## Abstract (web summary)

In a mouse model of spinal cord injury, the developmental stage of transplanted neural progenitor cells is shown to influence anatomical and behavioral outcomes after spinal cord injury

## Introduction

Spinal cord injury results in immediate and permanent loss of spinal cord neurons, frequently causing lifelong neurological dysfunction including but not limited to paralysis, loss of sensation, autonomic dysfunction, and chronic neuropathic pain^1–3^. Transplantation of neural progenitor cells is viewed as a promising neuronal replacement strategy with potential to regenerate neural circuits and improve functional outcomes following SCI^4,5^. Indeed, in the past few decades, there have been several clinical trials evaluating the therapeutic potential of neural stem- and progenitor cell transplantation for treatment of SCI in humans^6–11^. Despite advancement to clinical trials, further characterization of graft biology and therapeutic mechanism is sorely needed. For example, although it has been shown that spinal cord NPC grafts are populated with diverse neuronal subtypes^12–19^, it is still poorly understood how graft cellular composition influences regeneration of host axons and functional outcomes following SCI.

Over the past four decades, a great deal of knowledge has been gained from experimental rodent studies transplanting fetal rodent spinal cord NPCs^19^. These cells are exposed to normal developmental patterning cues and differentiate into multiple endogenous spinal cord neuronal subtypes following transplantation^12–18,20–23^, making them a gold standard cell source to characterize graft/host biology in cellular transplantation studies. A 1983 study by Reier, Perlow, and Guth was the first to demonstrate the survival and neurogenic potential of rat fetal spinal cord solid tissue grafts, derived from embryonic days 12 to 17 (E12–E17), following transplantation into the injured adult central nervous system (CNS)^24^. Due to their long-term survival and neural differentiation, E14–E15 embryos were concluded to be the “most optimal source” for spinal cord transplants compared to later-stage grafts^24^. Based on a literature survey of 70 fetal rodent spinal cord NPC transplantation studies, E14 rat spinal cord (developmentally equivalent to E12.5 in mouse^25^) remains the most commonly used source of NPC donor tissue, with 86.6% of these studies utilizing cells derived from this embryonic stage (Table 1). Despite this widespread use of a single developmental age of donor tissue, spinal cord neurogenesis occurs over a five-day period in the rodent^26,27^. Distinct populations of spinal cord neurons are born at different intervals within the period of neurogenesis, with varying abundances of the 11 progenitor populations over time^26^. This raises the possibility that transplantation of NPCs obtained from distinct days within the neurogenic period might produce grafts with varying neuronal subtype composition.

**Table 1:** Developmental stage of rodent embryos used as a source of donor spinal cord tissue in transplantation studies.

Developmental stage	Number of studies	Details
*Studies utilizing rat donor tissue*		
≤E13	6	Cultured multipotent neuroepithelial cells derived from E10.5 fetal spinal cord^17^ Solid pieces of E11-E13 fetal spinal cord^24,106–109^
E13.5–E14.5	58	Solid pieces of E14 fetal spinal cord^21,24,28,108,110–132^ Dissociated and cultured neural-restricted precursors, glial-restricted precursors, or cell suspensions derived from E13.5-E14.5 fetal spinal cord^14,16–18,133–142^ Dissociated (not cultured) E13.5-E14 fetal spinal cord^12,13,86,139,143–156^
≥E15	3	Solid pieces of E15-E17 fetal spinal cord^24,106,107^
*Studies utilizing mouse donor tissue*		
E12.0–E12.5	5	Dissociated E12-E12.5 fetal spinal cord^49,100,150,157,158^
≥E13	6	Dissociated and cultured cells derived from E13.5-P0 spinal cord^100,159–163^

Seventy experimental transplantation studies utilizing rodent fetal spinal cord-derived tissue are listed in this table. Studies utilizing transplantation of human cells, adult rodent stem cells, or cells obtained from fetal rodent tissues other than spinal cord are not included. Note that some studies utilized embryos of multiple ages, and those are duplicated in each applicable row.

We and others have previously shown that dorsal- or ventral-restricted populations of spinal cord NPCs impart distinct effects on host axon regeneration^12^ and respiratory function^28^ after SCI. Here, we explore how the developmental restriction of donor NPCs influences graft neuronal subtype composition, host axon regeneration into grafts, and recovery of sensorimotor function following SCI. Following the isolation of spinal cord NPC populations from E11.5, E12.5, and E13.5 mouse embryos, we assessed the abundances of distinct progenitor populations and spinal cord neuronal subtypes in vitro and in vivo. We also analyzed the effects of graft type on gliogenesis, graft axon outgrowth, and host axon regeneration into grafts. Finally, we determined the effects of NPC graft type on recovery of locomotor function and sensory function following transplantation into sites of thoracic SCI.

## Results

### Developmental stage of spinal cord neural progenitor cells affects the abundance of distinct progenitor populations and postmitotic cell populations in vitro

**Fig. 1:**
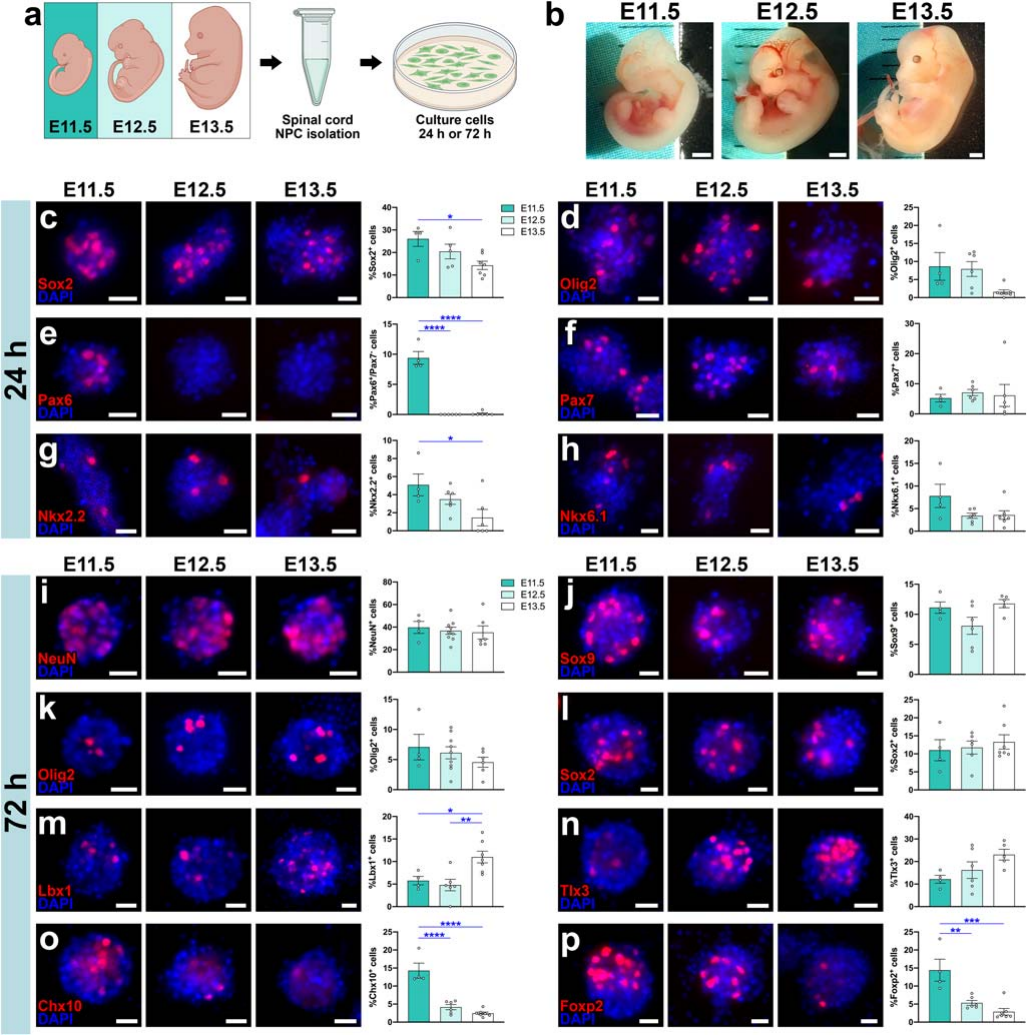
Characterization of spinal cord neural progenitor cells obtained from different developmental stage embryos. **a** Experimental design. Embryonic spinal cords were obtained from E11.5, E12.5, and E13.5 mouse embryos and NPCs were isolated from spinal cord tissue. Cells were cultured for either 24 or 72 h prior to fixation and immunostaining. Illustration created with BioRender.com. **b** Photographs of E11.5, E12.5, and E13.5 embryos. **c**–**h** Images of NPCs following 24 h of in vitro culture, immunolabeled for markers of neural progenitor populations **c** Sox2, **d** Olig2, **e** Pax6, **f** Pax7, **g** Nkx2.2, and **h** Nkx6.1. Graphs in panels **c**–**h** represent the percent of DAPI^+^ cells in each condition that express the corresponding NPC markers. *P < 0.05, **P < 0.01, ****P < 0.0001 by one-way ANOVA with Tukey’s multiple comparisons test. E11.5 (*n* = 4 wells), E12.5 (*n* = 5-6 wells), E13.5 (*n* = 6-7 wells). All data are mean ± SEM. **i**–**p** Images of NPCs following 72 h of in vitro culture, immunolabeled for **i** neuronal marker NeuN, **j** astroglial marker Sox9, **k** oligodendrocyte marker Olig2, **l** neural progenitor cell marker Sox2, and postmitotic neuronal subtype markers **m** Lbx1, **n** Tlx3, **o** Chx10, and **p** Foxp2. Graphs in panels **i**–**p** re**p**resent the percent of DAPI^+^ cells in each condition that express the corresponding NPC markers. *P < 0.05, **P < 0.01, ***P < 0.001, ****P < 0.0001 by one-way ANOVA with Tukey’s multiple comparisons test. E11.5 (*n* = 4 wells), E12.5 (*n* = 6–9 wells), E13.5 (*n* = 5–7 wells). All data are mean ± SEM. Scale bars = 1 mm (**b**), 20 μm (**c**–**p**). Source data are provided as a Source Data file. The experiments in panels **c**–**p** were performed twice with similar results.

Mouse spinal cord neurogenesis occurs from embryonic days E9.5 to E13.5. Relative abundances of the 11 cardinal spinal cord neural progenitor populations (dp1-dp6, p0-p3, pMN) shift over time during this neurogenic period^26,27,29^. Recent work has demonstrated that ventral progenitor populations are most abundant during early neurogenesis, and dorsal progenitors dominate in the later stages of neurogenesis^26^. The overall goal of our study is to determine how the transplantation of developmentally restricted NPC populations into sites of spinal cord injury affects graft cellular composition and integration with the injured host spinal cord. We first sought to characterize the cellular makeup of spinal cord isolates obtained from different days within the neurogenic period. We dissociated whole spinal cords from E11.5, E12.5, or E13.5 mouse embryos, then cultured the cell suspensions for either 24 or 72 h (Fig. 1a). For embryos younger than E11.5, we found that spinal cord tissue was difficult to separate from the surrounding meninges, so we were unable to obtain pure spinal cord cell preparations. Beyond size difference, embryos at these stages of development exhibited distinct morphological landmarks^30,31^. For example, forelimb buds of E11.5 embryos lack digit separation, whereas E12.5 embryos feature the first appearance of digit separation and digits of E13.5 embryos are more completely separated (Fig. 1b). We referred to these landmarks during the embryo harvest procedure and excluded the occasional embryos that appeared underdeveloped for their gestational age, compared to their littermates. Greater cell yields were obtained from later-stage embryos versus earlier-stage embryos, which is unsurprising given the difference in size of their spinal cords.

We first assessed immunohistochemical markers of distinct progenitor populations after spinal cord cell suspensions were cultured for 24 h in vitro. Diagram of transcription factor expression in spinal cord progenitors and postmitotic neurons is provided in Fig. S1. Sox2, a broad marker of neural stem- and progenitor cells in the CNS^32,33^, was detected in ~26.0% of E11.5 cells, 20.5% of E12.5 cells, and 14.3% of E13.5 cells, such that E13.5 cultures contained significantly fewer progenitors than E11.5 (*P* = 0.0227) (Fig. 1c). Cells expressing Olig2, a transcription factor restricted to the spinal pMN progenitor domain that gives rise to motor neurons and oligodendrocytes^34^, trended toward lower abundance in the E13.5 group compared to the other two groups (*P* = 0.0792 vs. E11.5; *P* = 0.0755 vs. E12.5; Fig. 1d). Trend analysis showed that the linear downward trend across groups was statistically significant [*F*(1,14) = 5.592, *P* = 0.0330]. The E11.5 group also contained significantly greater numbers of cells expressing Pax6, which is expressed in the dorsal and ventral (p0-p2) neural tube^35,36^, but not Pax7, compared to the other groups in which Pax6 was basically undetectable (*P* < 0.0001 vs. E12.5 and E13.5; Fig. 1e). However, there were no significant differences in the abundance of dorsal Pax7^+^ progenitors^35,37,38^ among groups (*P* = 0.8868; Fig. 1f). Finally, we analyzed the expression of Nkx2.2 and Nkx6.1, transcription factors whose expression are restricted to the p3 and p2/p3/pMN ventral progenitor domains, respectively^39–41^ (Fig. 1g, h). E13.5 cultures had significantly fewer Nkx2.2^+^ cells than E11.5 cultures (P = 0.0364), and these cultures also trended toward containing fewer Nkx6.1^+^ cells than E11.5 cultures; this linear downward trend was statistically significant [*F*(1,14) = 5.017, *P* = 0.0419]. Together, these results demonstrate that ventral spinal cord neural progenitor classes are more abundant in earlier-stage NPC cultures than later-stage cultures.

We next assessed markers of postmitotic cell populations after 72 h in vitro. We analyzed neurons (NeuN^+^), astrocytes (Sox9^+^), oligodendrocytes (Olig2^+^), and neural progenitors (Sox2^+^), and did not detect any significant differences among treatment groups in the abundance of these cells (Fig. 1i–l). We also quantified the abundance of dorsal spinal cord postmitotic cells. dILA/dILB interneurons are late-born cells within the superficial dorsal spinal cord laminae I-IV, and include Lbx1^+^ GABAergic dILA neurons and Tlx3^+^ glutamatergic dILB neurons^42^. We found that Lbx1^+^ dILA neurons were significantly more abundant in E13.5 cultures versus E11.5 (*P* = 0.0066) and E12.5 (*P* = 0.0394) cultures (Fig. 1m). There were no significant differences between groups in the abundance of Tlx3^+^ dILB neurons, although there was a statistically significant upward trend across groups [*F*(1,12) = 5.719, *P* = 0.0341; Fig. 1n]. Finally, we examined ventral SpIN populations. Chx10 is a marker of ventrally located, excitatory V2a SpINs^43,44^, and Foxp2 is a transcription factor expressed in a subset of V1 non-Renshaw SpINs in the ventral spinal cord, which first emerges at E11.0^45–47^. In both cases, we observed that E11.5 cultures contained significantly greater numbers of ventral V1 and V2a SpIN populations than E12.5 and E13.5 cultures (Foxp2: E11.5 vs E12.5, *P* = 0.0025; E11.5 vs. E13.5, *P* = 0.0002; Chx10: E11.5 vs. E12.5, *P* < 0.0001; E11.5 vs. E13.5, *P* < 0.0001; Fig. 1o, p). Together, these data demonstrate that NPCs obtained from mouse E11.5, E12.5, and E13.5 spinal cords contain a mix of dorsal and ventral progenitors, differentiate into a mix of neurons and glial cells, and yield varying abundances of dorsal and ventral spinal cord neuronal subtypes following culture in vitro. In addition, it is important to note that due to the ages of embryonic development from which these cells were obtained, the initial cell suspensions likely contain a mixture of NPCs and postmitotic neurons^26^. This is supported by the observation that not all cells express the NPC marker Sox2 after 24 h in culture (Fig. 1c). However, for the purposes of this study we will refer to the cell preparations as “NPCs”, because the progenitors are the cells that are most capable of survival and proliferation after transplantation, and therefore these cells make up the majority of the mature graft.

### Earlier-stage NPC grafts exhibit lower neuronal density and enhanced axon outgrowth versus later-stage grafts

**Fig. 2:**
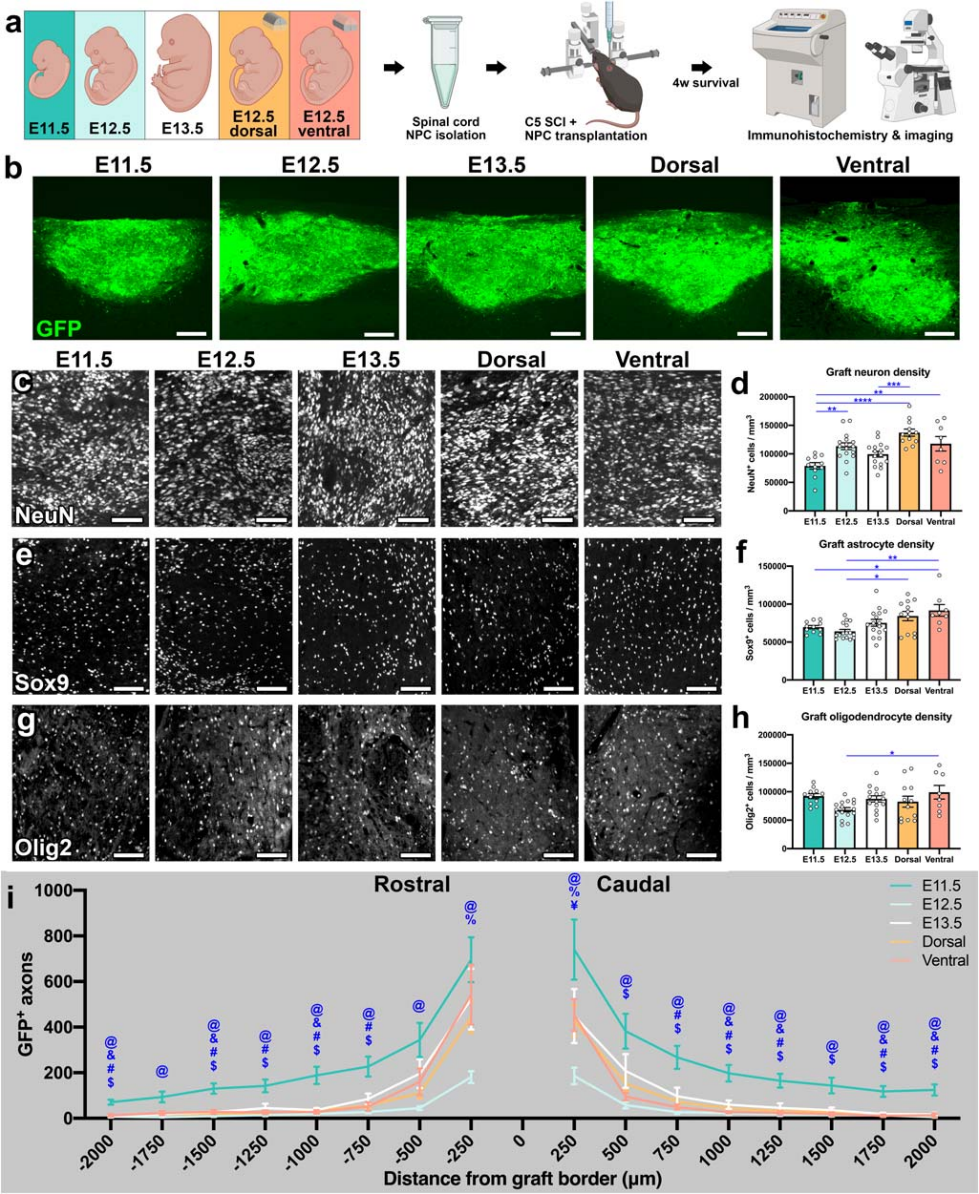
Developmental stage of donor NPCs does not substantially affect neurogenesis or gliogenesis, but earlier-stage grafts exhibit enhanced axon outgrowth. **a** Experimental design for the data presented in Figs. 2, 3, 5, and S1. Whole embryonic spinal cords were obtained from E11.5, E12.5, and E13.5 GFP^+^ mouse embryos, and NPCs were isolated from spinal cord tissue. Additional E12.5 spinal cords were subjected to dorsal/ventral dissection isolated prior to NPC isolation. Four weeks following NPC transplantation into cervical (C5) SCI, tissue was collected for immunohistochemical analysis. Illustration created with BioRender.com. **b** Representative images of GFP^+^ grafts. **c**, **e**, **g** Representative images showing the distributions of **c** NeuN^+^ neurons, **e** Sox9^+^ astrocytes, and **g** Olig2^+^ oligodendrocytes in grafts. **d**, **f**, **h** Quantification of **d** graft neuron density, **f** graft astrocyte density, and **h** graft oligodendrocyte density. *P < 0.05, **P < 0.01, ***P < 0.001, ****P < 0.0001 by one-way ANOVA + Tukey’s multiple comparisons test. **i** Quantification of graft-derived GFP^+^ axon outgrowth at 250-μm intervals rostral and caudal to the graft. Statistical significance for panel **i** is indicated by the following symbols: @ *P* < 0.05 for E11.5 vs. E12.5; & *P* < 0.05 for E11.5 vs. E13.5; # *P* < 0.05 for E11.5 vs. dorsal; $ *P* < 0.05 for E11.5 vs. ventral; % *P* < 0.05 for E12.5 vs. dorsal; ¥ *P* < 0.05 for E12.5 vs. ventral by two-way repeated measures ANOVA with Tukey’s multiple comparisons test. All data are mean ± SEM. E11.5 (*n* = 11); E12.5 (*n* = 16); E13.5 (*n* = 16 in **d**, **f**, **i**; *n* = 15 in **h**); dorsal (*n* = 12); ventral (*n* = 8). Scale bars = 200 μm (**b**), 100 μm (**c**, **e**, **g**). Source data are provided as a Source Data file. The experiments in panels **b**, **c**, **e**, and **g** were performed twice with similar results.

We next performed in vivo transplantation experiments to determine the overall effects of graft type on commonly assessed outcomes including neurogenesis, gliogenesis, and axon outgrowth. NPCs were isolated from whole spinal cords of E11.5, E12.5, or E13.5 GFP^+^ mouse embryos, as well as from the dorsal or ventral halves of E12.5 spinal cords; cells were transplanted into sites of acute C5 dorsal column SCI in adult, wild-type mice, and allowed to survive for 4 weeks (Fig. 2a). We included dorsal and ventral E12.5 NPC groups because we and others have previously shown that transplantation of dorsoventrally restricted rat spinal cord NPCs results in significant differences in graft cellular composition and immunoreactivity for specific axon fiber subtypes^12,23,48^; hence, this serves as a useful strategy for manipulating graft composition. At four weeks post-transplantation, all grafts exhibited strong GFP fluorescence and complete filling of the lesion site, with a typical degree of vascularization^49^ (Fig. 2b). Supplementary Movie 1 shows a 3D rendering of a GFP^+^ E12.5 graft at four weeks post-transplantation.

We first sought to determine whether the developmental stage of donor NPCs affected the density of neurons and glia in grafts. All grafts contained large numbers of neurons (Fig. 2c), but we found that E11.5 grafts had significantly fewer neurons per unit volume compared to E12.5 grafts (*P* = 0.0039), dorsal grafts (*P* < 0.0001), and ventral grafts (*P* = 0.0068; Fig. 2d). We next quantified numbers of Sox9^+^ astrocytes within grafts, and did not detect significant differences among the three different developmentally restricted NPC grafts, although ventral grafts had significantly more astrocytes per unit volume than E11.5 grafts (*P* = 0.0370) and E12.5 grafts (*P* = 0.0020; Fig. 2e, f). This may reflect the fact that gliogenesis exclusively occurs in ventral spinal progenitor domains^50–55^. Similarly, there were no significant inter-group differences in graft Olig2^+^ oligodendrocyte density, except that ventral grafts contained more cells than E12.5 (whole) grafts (*P* = 0.0322; Fig. 2g, h). We also quantified the extension of GFP^+^ graft-derived axons in the host spinal cord up to a total distance of 2 mm in the rostral and caudal directions. Representative images of axon outgrowth are shown in Fig. S2. E11.5 grafts exhibited significantly greater axon outgrowth than E12.5 grafts at every interval measured (Fig. 2i, Supp. Fig. 2). In addition, axon outgrowth of E11.5 grafts was significantly greater than ventral grafts at 12 of 16 intervals, significantly greater than dorsal grafts at 10 of 16 intervals, and significantly greater than E13.5 grafts at 7 of 16 intervals. Collectively, these results suggest that E11.5 NPC grafts extend more axons for greater distances, despite decreased neuronal density compared to other graft types.

### Developmental stage of donor NPCs significantly influences graft neuronal subtype composition

**Fig. 3:**
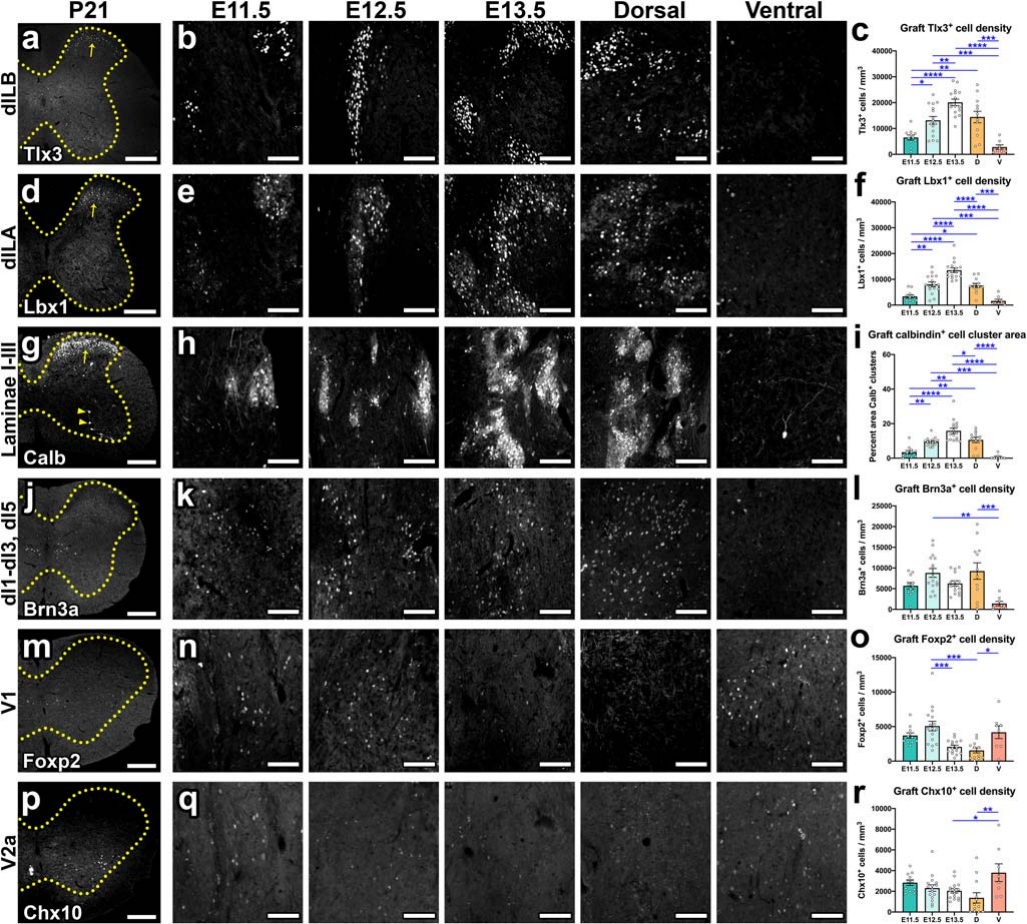
Developmental stage of donor NPCs significantly influences graft neuronal subtype composition. **a**, **d**, **g**, **j**, **m**, **p** Distribution of cell type-specific markers in intact P21 mouse spinal cord. **b**, **e**, **h**, **k**, **n**, **q** Images of NPC grafts at 4 weeks post-transplantation into sites of cervical SCI. Images within the same column in rows **b** and **e**, as well as rows **n** and **q**, are different fluorescent channels of the same tissue section. **c**, **f**, **i**, **l**, **o**, **r** Quantification of the abundance of **c** Tlx3^+^, **f** Lbx1^+^, **i** clustered calbindin^+^, **l** Brn3a^+^, **o** Foxp2^+^, and **r** Chx10^+^ interneurons in NPC grafts. **e** Note the different populations of clustered calbindin^+^ neurons (arrow) and non-clustered, larger calbindin^+^ neurons (arrowheads). *P < 0.05, **P < 0.01, ***P < 0.001, ****P < 0.0001 by one-way ANOVA with Tukey’s multiple comparisons test. E11.5 (*n* = 11); E12.5 (*n* = 16); E13.5 (*n* = 16); dorsal (*n* = 12); ventral (*n* = 8). All data are mean ± SEM. Scale bars = 250 μm (**a**, **d**, **g**, **j**, **m**, **p**); 100 μm (**b**, **e**, **h**, **k**, **n**, **q**). Source data are provided as a Source Data file. The experiments in panels **a**, **b**, **d**, **e**, **g**, **h**, **j**, **k**, **m**, **n**, **p**, and **q** were performed twice with similar results.

Although the full extent of spinal cord NPC graft cellular diversity is not yet appreciated, grafts have been shown to contain diverse neuronal subtypes^19^. This includes dorsal and ventral spinal interneurons (SpINs), which encompass early-born and late-born populations^27,56–59^. The dorsal horn contains the late-born dILA and dILB classes of neurons, which emerge from the dorsal progenitor domain from E12 to E14.5^60–62^. We performed immunohistochemistry to examine the distribution of dorsal dILA/dILB spinal cord interneurons in NPC grafts at 4 weeks post-transplantation into sites of C5 dorsal column SCI. We observed that E13.5 grafts contained significantly greater abundances of Tlx3^+^ dILB neurons than all other groups except E12.5 dorsal (*P* < 0.0001 vs. E11.5; *P* = 0.0048 vs. E12.5; *P* < 0.0001 vs. ventral; Fig. 3a–c). For Lbx1^+^ dILA neurons, E13.5 grafts contained greater density of these cells than all other groups (*P* < 0.0001 vs. all groups; Fig. 3d–f). As expected^12^, dorsal grafts were also significantly enriched for both cell types compared to ventral grafts (Tlx3: *P* = 0.0001, Lbx1: *P* = 0.0008). These results are consistent with the late-born, dorsal origin of dILA/dILB neurons, demonstrating that not only the regional origin but also the developmental timing of NPCs collected for transplantation affect graft cellular composition.

Clustered calbindin^+^ (Calb) neurons residing in dorsal horn laminae I–III are a heterogeneous population of cells with diverse projections, some of which are derived from Gbx2^+^ spinal cord progenitors^63–68^ (Fig. 3g). We previously demonstrated that clustered calretinin^+^ neurons (a distinct heterogeneous population in laminae I/II) could be enriched in grafts by transplantation of dorsally restricted rat spinal cord NPCs^12^. Similarly, we observed that dorsal grafts contained significantly greater area occupied by clustered Calb^+^ neurons compared to ventral grafts (*P* < 0.0001). Furthermore, E13.5 grafts contained significantly greater Calb^+^ cluster area than every other group (*P* < 0.0001 vs. E11.5; *P* = 0.0011 vs. E12.5; *P* = 0.0191 vs. dorsal; *P* < 0.0001 vs. ventral; Fig. 3h, i). This suggests that dorsal horn Calb^+^ neurons emerge from late-born lineages, possibly a subset of the dILA/dILB populations. Brn3a (Pou4f1) is a transcription factor expressed exclusively in dorsal spinal cord SpINs including class A dorsal spinal cord neurons (dI1–dI3) and class B dI5 neurons, which are all born around E10.5–10.75^62^, as well as late-born dILA neurons^69^ (Fig. 3j). As expected, these dorsal neuron populations were significantly enriched in dorsal grafts compared to ventral grafts (*P* = 0.0010) (Fig. 3k, l). However, we did not observe any significant differences in Brn3a^+^ neuron density among the three different developmentally restricted graft types. This may reflect expression of Brn3a in a mixture of early-born (dI1–dI3, dI5) and late-born (dILA) cell types in grafts.

We next assessed the abundances of ventral SpIN populations in grafts. We previously showed that Foxp2^+^ V1 SpINs were enriched in ventral rat spinal cord NPC grafts^12^, and we confirmed this observation in the current mouse NPC transplantation study (*P* = 0.0311 for dorsal vs. ventral) (Fig. 3m–o). Among the developmentally restricted graft types, E12.5 grafts contained significantly more Foxp2^+^ neurons compared to E13.5 grafts (*P* = 0.0004), reflecting the early birth date of this population. Finally, we quantified the abundance of Chx10^+^ V2a SpINs (Fig. 3p). The abundance of Chx10-immunoreactive cells in grafts was lower across the board, for all graft types, compared to the abundances of other cell populations (Fig. 3q, r). As expected, we found that ventral NPC grafts contained higher density of Chx10^+^ neurons than dorsal grafts (*P* = 0.0033). However, there were no significant differences in Chx10^+^ neuron density among developmentally restricted graft types.

Not all neuronal subtypes that we analyzed showed differential abundance among developmentally restricted graft types placed into sites of C5 dorsal column SCI. Bhlhb5 is a transcription factor that is expressed in dI6, V1, and V2 interneurons in the intermediate gray matter as well as a subset of late-born dorsal interneurons within the superficial dorsal horn laminae^70,71^ (Fig. S3a). Although numbers of Bhlhb5^+^ interneurons did not significantly differ among E11.5, E12.5, and E13.5 grafts, both E11.5 and E12.5 grafts contained significantly more Bhlhb5^+^ neurons versus dorsal grafts (*P* = 0.0365 for E11.5 vs dorsal; *P* = 0.0224 for E12.5 vs. dorsal; Fig. S3b–c). We next examined the expression of the calcium-binding proteins, calbindin and parvalbumin (Parv), in graft-derived neurons. Aside from the clustered band of small-diameter Calb^+^ neurons that is present in laminae I–III of the dorsal horn (Fig. 3g), there are also non-clustered, larger-diameter Calb^+^ neurons in the intermediate and ventral gray matter (Fig. S3d). Some of these Calb^+^ neurons are Renshaw cells that also express Parv^72,73^ (Fig. S3j). In addition, there are also Parv^+^/Calb^−^ neurons in the dorsal and ventral spinal cord gray matter^67,73,74^, although their functional roles are not well characterized (Fig. S3g). We did not observe differences in the abundances of either Calb^+^/Parv^−^ cells, Parv^+^/Calb^−^ cells, or Renshaw cells among developmentally restricted graft types, although Calb^+^/Parv^−^ neurons were least abundant in ventral grafts (Fig. S3e–l).

Many SCI transplantation studies utilize rat tissue as a source of donor NPCs; in these studies, E14 embryos are the most commonly utilized source of donor cells (Table 1). Rat E14 embryos are developmentally equivalent to E12.5 mouse embryos (~52–55 somite stage)^25,75^. We therefore compared abundances of four SpIN subtypes in grafts of spinal cord NPCs derived from E13, E14, and E15 rat embryos and transplanted into sites of C5 dorsal column SCI (Fig. S4). Similar to our observations with mouse NPC grafts, we found that dorsal SpIN populations (Tlx3^+^ dILB neurons and Lbx1^+^ dILA neurons) were enriched in later-stage grafts compared to earlier-stage grafts (Fig. S4a–d). We did not observe any significant differences in the abundance of Foxp2^+^ V1 interneurons among groups, although there was a non-significant trend toward reduced abundance in E15 grafts (Fig. S4e–f). Ventral Chx10^+^ V2a neurons were most abundant in earlier-stage E13 grafts (Fig. S4g–h). Hence, transplantation of developmentally restricted NPCs is sufficient to modulate graft neuronal composition for rat as well as mouse embryonic spinal cord NPC grafts.

### Earlier-stage grafts are enriched for V2a neurons and Group-Z cells, and later-stage grafts are enriched for Group-N cells

**Fig. 4:**
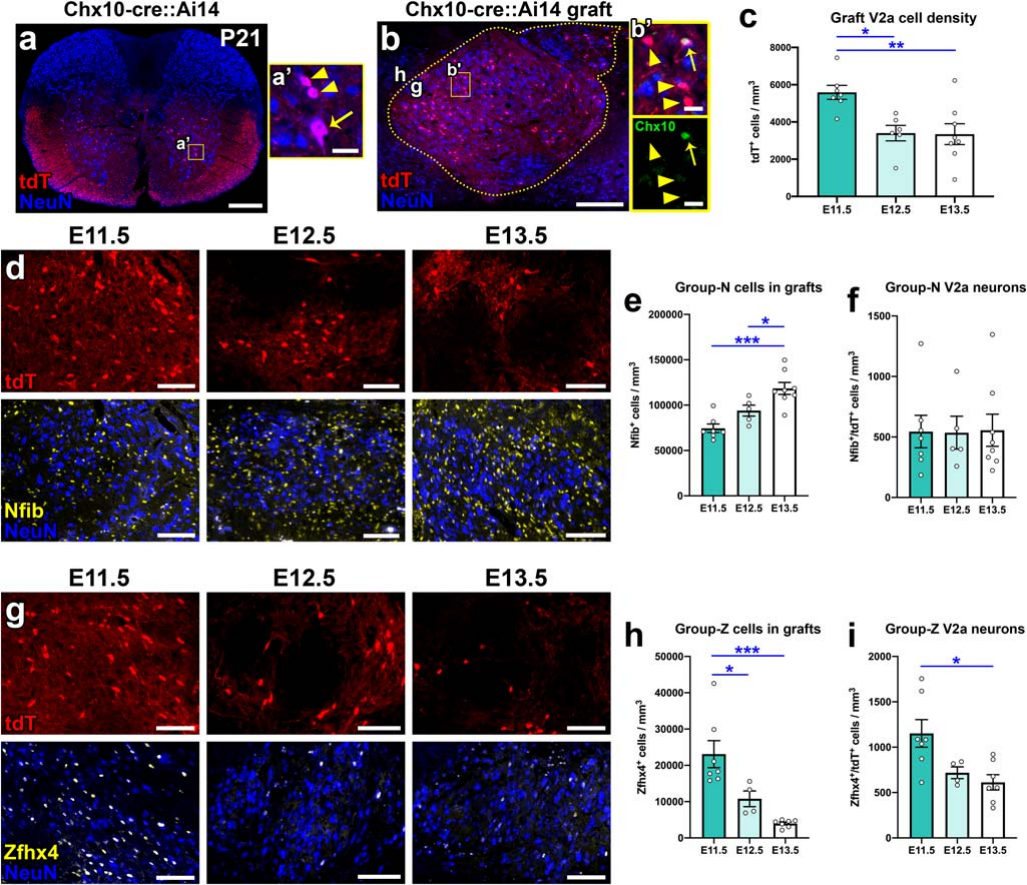
Differential abundance of V2a interneurons, Group-N cells, and Group-Z cells in grafts. **a** Cervical (C3) spinal cord section of a P21 Chx10-cre::Ai14 mouse, with tdTomato expression in V2a interneurons. **a’** Inset shows two small-diameter V2a interneurons (arrowheads) and one larger-diameter interneuron (arrow). **b** Image of E11.5 Chx10-cre::Ai14 NPC graft at 4 weeks post-transplantation (h, host; g, graft). Graft is outlined with dotted lines. **b’** Inset shows V2a interneuron in graft that expresses Chx10 (arrow) and other V2a interneurons that do not express detectable levels of Chx10 (arrowheads). **c** Quantification of the total numbers of tdTomato^+^ V2a interneurons in Chx10-cre::Ai14 NPC grafts. **d**, **g** Images of Chx10-cre::Ai14 grafts immunolabeled for **d** Group-N neuronal marker Nfib and **g** Group-Z neuronal marker Zfhx4. **e**, **h** Quantifications of **e** Group N and **h** Group Z cells in grafts. **f**, **i** Quantifications of **f** Nfib^+^ V2a interneurons and **i** Zfhx4^+^ V2a interneurons in grafts. *P < 0.05, **P < 0.01, ***P < 0.001 by one-way ANOVA with Tukey’s multiple comparisons test. E11.5 (*n* = 7); E12.5 (*n* = 4–5); E13.5 (*n* = 7–8). All data are mean ± SEM. Scale bars = 250 μm (**a**, **b**), 100 μm (**d**, **g**), 25 μm (**a’**, **b’**). Source data are provided as a Source Data file. The experiments in panels **a**, **b**, **d**, and **g** were performed twice with similar results.

V2a SpINs are an important premotor population that drive motor neuron firing and are involved in diverse motor functions including locomotion^76,77^, skilled forelimb reaching^76,78^, and respiration^79^. V2a SpINs have been shown to undergo plastic changes and contribute to functional recovery following SCI^80,81^; notably, Zholudeva and colleagues demonstrated that transplanted V2a SpINs improve recovery of respiratory function after SCI^14^. Hence, the V2a SpIN population is an attractive potential target for promoting motor recovery after SCI. In the postnatal spinal cord, type I V2a SpINs express high levels of Chx10, while type II V2a SpINs downregulate Chx10 expression^76^. We therefore speculated that quantification of graft V2a SpINs based on Chx10 immunoreactivity (Fig. 3q, r) may yield an underestimation of the true numbers of V2a neurons in grafts. To address this, we generated grafts derived from spinal cord NPCs of Chx10-cre::Ai14 embryos, in which all donor V2a SpINs express the tdTomato reporter^78,82^. As expected, spinal cords of postnatal Chx10-cre::Ai14 mice exhibited tdTomato expression that was restricted to V2a SpINs, which are located in the ventral spinal cord gray matter, as well as their axons, which extend throughout the ventral and lateral spinal cord white matter (Fig. 4a). Grafts of Chx10-cre::Ai14 NPCs also contained V2a SpINs that expressed tdTomato (Fig. 4b). We quantified the numbers of tdT^+^/NeuN^+^ V2a SpINs in grafts placed into sites of C5 dorsal column SCI and found that E11.5 grafts had the highest density of V2a SpINs, significantly greater than both E12.5 grafts (*P* = 0.0155) and E13.5 grafts (*P* = 0.0078) (Fig. 4c). These data confirm that analyzing Chx10 immunoreactivity alone is not an adequate approach for quantifying total V2a SpINs in NPC grafts; indeed, reporter cell labeling identified a two-to-three-fold increase in V2a SpIN abundance.

Beyond spinal cord SpINs born from different progenitor domains, distinct regional classes of spinal cord SpINs also have different birthdates^26,29,83^. Osseward and colleagues recently characterized subpopulations of ventrally located spinal cord motor control neurons born on different birth dates, identifying distinctions between a laterally located “Group-Z” class (enriched for Zfhx3, Zfhx4, and Foxp2) born around E10.5, and a medially located “Group-N” class (enriched for Nfib, NeuroD2, and Prox1) born around E13.5^83^. This classification subdivides the cardinal classes of neurons, including V2a SpINs^76^, into local (Group-N) and projection (Group-Z) neurons. We therefore assessed whether these cell types were differentially distributed in E11.5, E12.5, and E13.5 grafts. In Chx10-cre::Ai14 grafts, we observed that not all Group-N cells were neurons; many non-neuronal cells were also labeled (Fig. 4d). Through quantification of all Nfib^+^ Group-N cells in each graft type, we found that E13.5 grafts contained significantly more of these cells than the other two graft types (*P* = 0.0001 vs. E11.5, *P* = 0.0340 vs. E12.5; Fig. 4e). This is consistent with the later birthdate of Group-N neurons in the normal developing spinal cord^83^. However, there were only scant numbers of Nfib^+^ V2a neurons in all graft types, with this population representing <1% of total graft neurons (Fig. 4f). In contrast, we detected significantly greater numbers of Zfhx4^+^ neurons in E11.5 grafts than the other groups (*P* = 0.0230 vs. E12.5, *P* = 0.0002 vs. E13.5; Fig. 4g, h). Likewise, there were significantly more Group-Z (Zfhx4^+^) V2a neurons in E11.5 grafts compared with E13.5 grafts (*P* = 0.0105; Fig. 4i). Collectively, these findings illustrate that NPC grafts generated from distinct donor cell stages contain diversity not only with regard to the cardinal classes of neurons contained within (Fig. 3, S3, S4), but also with regard to the “N-Z Division”, which determines neurons’ projections and mediolateral positions^83^.

### Graft type significantly influences regeneration of descending and afferent host axon populations

**Fig. 5:**
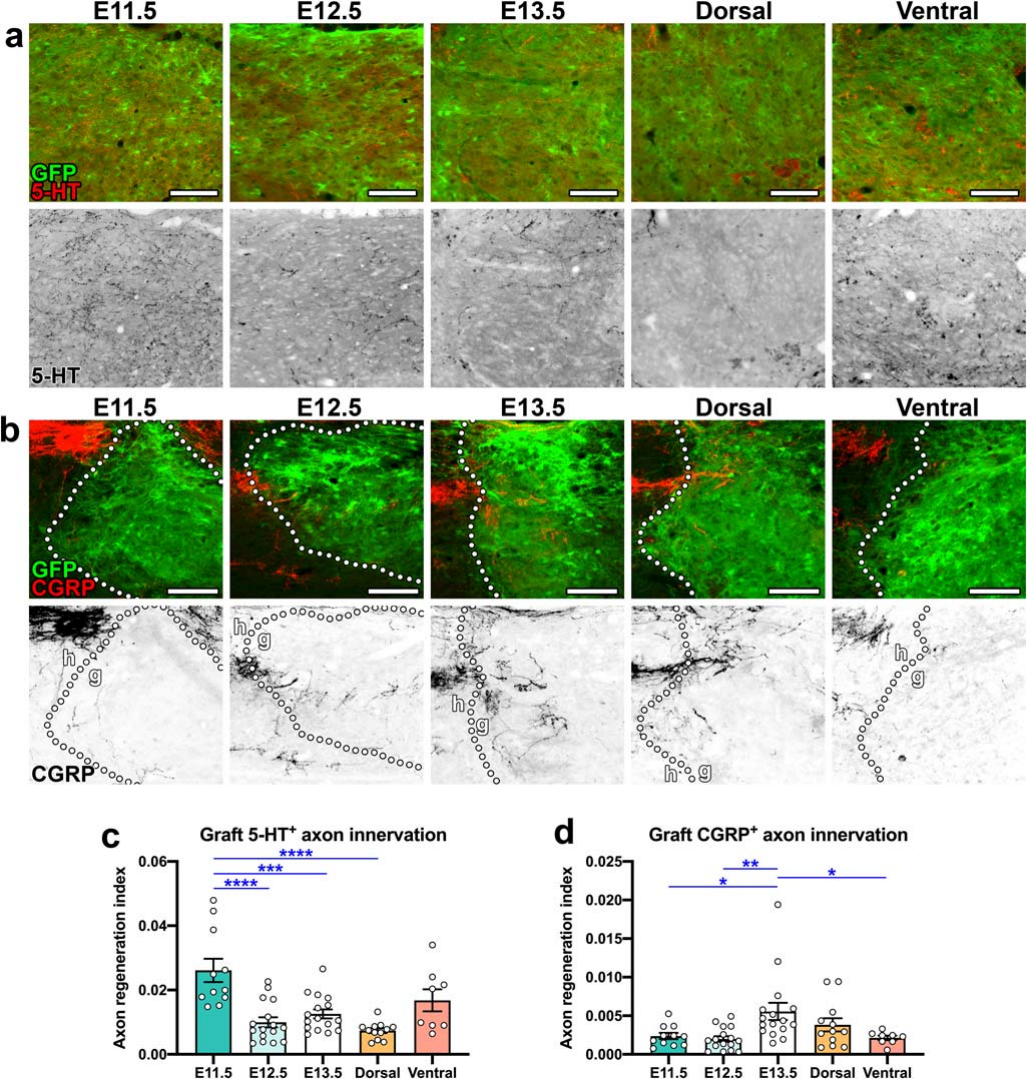
Host serotonergic axons preferentially regenerate into earlier-stage grafts and host nociceptive axons preferentially regenerate into later-stage grafts. Images of GFP^+^ grafts at 4 weeks post-transplantation, showing regeneration of **a** host 5-HT^+^ axons and **b** host CGRP^+^ axons into grafts. The bottom row of each panel shows axons in grayscale. **b** Host CGRP^+^ fibers are shown at the upper left of each image; each field of view contains the graft/host border (dotted lines) with point of entry of CGRP^+^ axons into grafts (**g**). **c**, **d** Quantification of **c** 5-HT^+^ axon density and **d** CGRP^+^ axon density within grafts. *P < 0.05, **P < 0.01, ***P < 0.001, ****P < 0.0001 by one-way ANOVA with Tukey’s multiple comparisons test. E11.5 (*n* = 11); E12.5 (*n* = 16); E13.5 (*n* = 16); dorsal (*n* = 12); ventral (*n* = 8). All data are mean ± SEM. Scale bars = 100 μm. Source data are provided as a Source Data file. The experiments in panels a and b were performed twice with similar results.

Regeneration of injured host axons into NPC grafts is a critical requirement for establishing new graft/host neural relays following SCI^4^. We previously found that modulating the cellular composition of rat spinal cord NPC grafts through dorsal/ventral restriction significantly influenced the extent of host corticospinal and nociceptive axon regeneration into grafts^12^. We next sought to examine regeneration of functionally important host axon populations into each graft type. Serotonergic (5-HT^+^) neurons in the brainstem that project to the spinal cord have been shown to modulate sensory, motor, and autonomic functions; these projections have been shown to modulate locomotor functional recovery following SCI^84,85^. It has previously been shown that 5-HT^+^ axons regenerate spontaneously into NPC grafts^86^. We analyzed 5-HT^+^ axon regeneration into GFP^+^ NPC grafts at 4 weeks post-transplantation into sites of C5 dorsal column SCI, and found that E11.5 grafts supported significantly greater serotonergic axon regeneration versus E12.5 grafts (*P* < 0.0001), E13.5 grafts (*P* = 0.0002), and dorsal grafts (*P* < 0.0001) (Fig. 5a, c). We next assessed growth of nociceptive axons into grafts. We previously found that peptidergic C-fibers expressing calcitonin gene-related peptide (CGRP) regenerate preferentially into laminae I/II “dorsal horn-like” domains of NPC grafts^12^. Given that E13.5 grafts were most highly enriched in laminae I/II dorsal horn neurons (Fig. 3a–i), we predicted that this group would exhibit the most extensive CGRP^+^ fiber regeneration. Indeed, we found that E13.5 grafts were the most densely innervated by CGRP^+^ axons, with significantly greater regeneration versus E11.5 (*P* = 0.380), E12.5 (*P* = 0.0053), and ventral grafts (*P* = 0.0475; Fig. 5b, d). Together, these data demonstrate that the cellular composition of NPC grafts, with specific regard to the diversity of neuronal subtypes contained within, significantly influences the degree to which host axon populations regenerate into the grafts.

### Developmental stage of donor NPCs significantly influences sensory, but not locomotor, behavioral outcomes

**Fig. 6:**
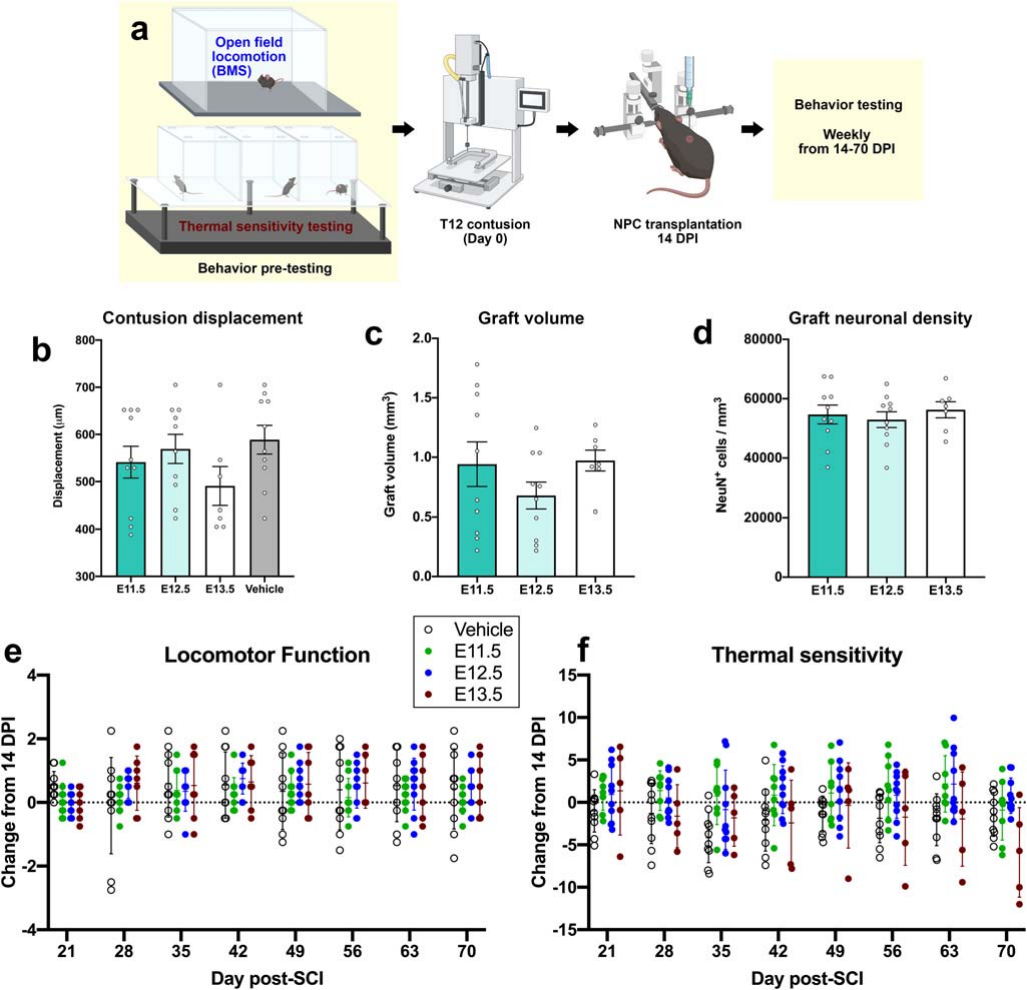
Graft type significantly influences sensory but not locomotor functional recovery after spinal cord injury. **a** Experimental design. Adult wild-type mice received baseline behavior testing in the open field and the Hargreaves thermal sensitivity testing apparatus. T12 contusion was delivered on Day 0, and transplantation of E11.5, E12.5, or E13.5 NPCs or vehicle was performed on Day 14 post-injury (DPI). Behavioral testing was conducted until 70 DPI. Illustration created with BioRender.com. **b** Impactor probe displacement values for each animal during the SCI surgery. There are no significant differences among groups as assessed by one-way ANOVA + Tukey’s multiple comparisons test. **c** Graft volume for NPC grafts placed into sites of thoracic contusion SCI. Vehicle (*n* = 10); E11.5 (*n* = 10); E12.5 (*n* = 10); E13.5 (*n* = 7). **d** Graft neuronal density for NPC grafts placed into sites of thoracic contusion SCI. Vehicle (*n* = 10); E11.5 (*n* = 10); E12.5 (*n* = 10); E13.5 (*n* = 7). **e** Open field locomotor (BMS) scores for each group at 21 DPI through 70 DPI, expressed as change from 14 DPI. There are no significant differences among groups as assessed by two-way repeated measures ANOVA. Vehicle (*n* = 10); E11.5 (*n* = 10); E12.5 (*n* = 10); E13.5 (*n* = 7). **f** Thermal sensitivity scores, expressed as change from 14 DPI withdrawal latencies, for each group from 14 DPI to 70 DPI. *P* = 0.0056 for group × time interaction (E11.5, E12.5, and E13.5) by one-way repeated measures ANOVA. Vehicle (*n* = 10); E11.5 (*n* = 8); E12.5 (*n* = 10); E13.5 (*n* = 5). All data are mean ± SEM. Source data are provided as a Source Data file.

We next performed a chronic behavioral experiment to ascertain the contribution of each graft type to recovery of hindlimb motor and sensory function in a clinically relevant SCI model. Mice received a moderate T12 thoracic contusion followed by transplantation of E11.5, E12.5, or E13.5 NPCs at 14 days post-SCI (Fig. 6a). Day 14 BMS scores were collected immediately prior to grafting. Displacement values for the spinal contusion injury were variable among subjects, but group mean scores did not differ significantly from one another (*P* = 0.2574; Fig. 6b). Graft volume and graft neuronal density also did not differ significantly among treatment group (Fig. 6c, d). Hindlimb motor function was assessed using the Basso Mouse Scale (BMS) for Locomotion^87^. Prior to NPC transplantation at 14 DPI, mice in all groups recovered to a BMS score of ~1–2, with no significant differences between groups (Fig. 6e). Over the 8 weeks that elapsed following cell transplantation, we did not observe any significant differences in hindlimb locomotor scores among any of the treatment groups (Fig. 6e). This demonstrates that NPC transplantation did not affect motor functional recovery in this injury model.

We also measured sensory function using the Hargreaves thermal sensitivity test. Analysis of all groups led to the identification of a group × time interaction, indicating that the change across days depended on group assignment (*P* = 0.001; Fig. 6f). To further explore the nature of the group × time interaction, an additional analysis was performed limited to the three groups that received implants. This analysis revealed that the change in thermal reactivity observed across days varied by graft type (F(14, 126) = 2.382, *P* = 0.0056). This effect emerged because mice in the E13.5 condition became more responsive across days while mice in the other two conditions showed the opposite trend. This suggests that a delayed effect of E13.5 NPC transplantation exacerbated pain-associated behaviors in this treatment group, possibly due to plasticity of graft/host neural circuits.

## Discussion

In this study, we have shown that the specific age of development from which embryonic rodent NPCs are obtained significantly influences graft cellular composition, graft axon outgrowth, host axon regeneration, and sensory behavioral outcomes following transplantation into the lesioned spinal cord. These findings have multiple implications for experimental SCI/transplantation studies. First, researchers utilizing primary rodent cells as a source of NPCs should carefully consider the age of embryo used in order to best suit the experimental objectives. For example, studies focusing on studying integration of grafts into host motor circuitry might utilize earlier-stage NPC grafts enriched for ventral premotor interneurons. For researchers using human pluripotent stem cell-derived neural stem and progenitor cells, it will be important to consider how grafts differentiate into distinct neuronal subtypes, given our findings that graft cellular composition influences anatomical and functional outcomes. As we learn more about the functional contributions of individual neuronal subtypes to motor functional recovery, it should eventually be possible to use modern directed differentiation techniques to generate customized NPC grafts enriched for cell types of interest.

There are some technical limitations to the use of E11.5 mouse embryos as donors. E11.5 spinal cords are more difficult to isolate and yield fewer cells compared to later embryonic stages. Given that mouse neurogenesis begins at about E9.5, it would be interesting to extend this study to include even earlier embryonic ages; however, isolation of the developing embryonic spinal cord while avoiding contamination from surrounding mesodermal tissue will be daunting for ages earlier than E11.5. Given recent advances in the development of mouse and human spinal cord organoids in vitro^88–90^, perhaps engineering earlier-stage neural tube tissue in this manner could address feasibility issues of isolating younger embryonic spinal cords.

Here, we analyzed cultured NPCs at 24 and 72 h in vitro to gain an appreciation for the types of cells that are initially being transplanted in our in vivo studies. However, we note that differences in abundance of cell types in culture do not necessarily accurately predict or reflect the abundances of the corresponding postmitotic cells in the mature graft. Delile and colleagues showed that there are differences in the abundances of distinct progenitor classes in the developing mouse neural tube during the 5 days of spinal cord neurogenesis^26^. These cells are likely proliferating to different degrees following transplantation into the hostile SCI lesion site. Whether some subtypes of progenitors are more likely than others to survive transplantation and proliferate in vivo is still unclear. In addition, this study has utilized immunohistochemistry to visualize markers of progenitor and neuronal subtypes that have previously been described in studies of the intact, developing spinal cord. We still lack a comprehensive understanding of NPC graft cellular diversity, and future work to perform unbiased single-cell transcriptional profiling of mouse and human cell grafts will be tremendously informative for grating studies.

In grafts placed into sites of SCI in vivo, we observed significant differences in the abundance of multiple neuronal subtypes among grafts of different developmental NPC stages. For example, ventral spinal cord V1 and V2a interneuron populations were enriched in earlier-stage grafts, and dorsal horn neuronal populations (dILA, dILB, laminae I-III calbindin^+^ clusters) were enriched in later-stage grafts. These observations closely reflect the differences in subtype abundance among E11.5, E12.5, and E13.5 NPCs that we observed after 72 h culture in vitro. Upon analyzing the distribution of distinct NPC lineages in vitro, we found that ventral progenitors (Pax6^+^ and Nkx2.2^+^ progenitors) were enriched in earlier-stage cells. Collectively, these findings corroborate what is known about the abundances of distinct dorsal and ventral spinal cord NPC populations during the neurogenic period; generally, that ventral progenitors are most proliferative at E10-E11 and dorsal progenitors are most proliferative at later stages^26^. Together, our data provide evidence that NPC cell fates are determined intrinsically during spinal cord development, prior to isolation and culture or transplantation of these cells, and that these fates are retained regardless of whether they are cultured in vitro or transplanted in vivo. This is unsurprising, given earlier work in the field of fetal spinal cord transplantation showing that fetal grafts placed into heterotopic environments (e.g., the brain, the anterior chamber of the eye) retained morphological characteristics of the adult spinal cord^20,21,24,91^.

We analyzed abundances of Group-N and Group-Z cells in grafts; these are recently described classifications of spinal cord neurons that go beyond the cardinal classes of neurons, dividing them into their “motor-sensory, local-long range, and excitatory-inhibitory features”^83^. Consistent with the findings of that study, here we observed greater abundance of the late-born Group-N neurons in later-stage E13.5 grafts, and greater abundances of the early-born Group-Z neurons in the earlier-stage E11.5 grafts. One hallmark of Group-Z neurons described by Osseward et al. is that they are projection neurons with long-distance axon projects to the thalamus, cerebellum, and brainstem. Here, we observed greatest axon outgrowth in E11.5 grafts, despite these grafts having a lower neuronal density than E12.5 grafts, suggesting that there is greater axon outgrowth per neuron in the E11.5 group. We speculate that this could potentially be attributed to the enrichment of these grafts for long-distance projection neurons, such as Group-Z projection neurons. Further work is needed to explore the subtypes of neurons that send long-distance projections in grafts, as these may be critical for relaying motor signals to the caudal spinal cord. We also observed differential regeneration of 5-HT^+^ and CGRP^+^ host axons into grafts. Given the importance of these axons in motor and sensory function in the intact spinal cord, respectively, this suggests that earlier-stage grafts may be better suited to studies of graft integration with motor circuits, and later-stage grafts might be better suited to studies of graft integration with sensory or pain circuits. We did not perform corticospinal tracing in this study, but it would be interesting to how graft cellular composition influences regeneration of this and other functionally important axon projections into grafts after SCI.

It is important to note that we did not observe any locomotor functional improvement with any of the graft types. This is in contrast to previous reports that NPC transplantation significantly improved locomotor performance after thoracic SCI^86,92–98^. It is unlikely that this is due to poor graft survival or transplantation of insufficient numbers of cells, because all subjects exhibited large surviving grafts with equivalent density of neurons. Our failure to observe motor functional improvement may be attributed to several factors such as the use of a different SCI model or different cell source than previous studies, the subjectivity of rodent open field locomotor scoring scales, or other yet-unappreciated biological factors. In normal development, new synaptic connections are stabilized and strengthened through activity. We hypothesize that NPC grafts will likewise not improve motor function after SCI unless use-dependent training is employed to shape the graft/host neural circuits that spontaneously form after transplantation. It may be unreasonable to expect grafts to integrate into and functionally modulate locomotor circuits if they are not ‘trained’ to do so. Ongoing work in our lab is examining the role of rehabilitation training in shaping graft/host synaptic connectivity and functional relays. Our present findings also suggest that grafts enriched with dorsal horn sensory processing interneurons might exacerbate sensory dysfunction following SCI. The mechanisms underlying this are unclear, but we show that CGRP^+^ axon regeneration into these grafts is also increased. It is possible that the enrichment of these grafts for “dorsal horn-like domains”^12^ may act as a ‘lure’ for aberrant sprouting nociceptive fibers, potentially affecting sensory neurotransmission in a way that gradually increases thermal hypersensitivity. Further study is needed to explore this possibility.

In conclusion, there is much that remains unclear with regard to the molecular and cellular mechanisms by which different graft types support different degrees of axon outgrowth, host axon regeneration, or functional recovery. Clearly, more work is needed to understand how NPC grafts synaptically and functionally integrate into host spinal cord, in order to build more therapeutic grafts that can robustly and reproducibly improve human neurological functions in the future.

## Methods

### Ethics statement

Animal experiments were performed in stringent compliance with the *NIH Guidelines for Animal Care and Use of Laboratory Animals*. All experiments were approved by the Texas A&M University Institutional Animal Care and Use Committee. All efforts were made to minimize pain and distress.

### Animals

A total of *n* = 310 mice were used for this study, including *n* = 198 8–10 week-old C57BL/6J mice (#000664, Jackson Laboratories; *n* = 76 males and *n* = 122 females), *n* = 4 P21 male and female C57BL/6 mice, *n* = 30 3–8 month-old male homozygous GFP mice [C57BL/6-Tg(CAG-EGFP)131Osb/LeySopJ; #006567, Jackson Laboratories], *n* = 18 3–8 month-old male homozygous Syn1-cre mice [B6.Cg-Tg(Syn1-cre)671Jxm/J, #003966, Jackson Laboratories], *n* = 24 8–10 week-old homozygous female RɸGT mice [B6;129P2-*Gt(ROSA)26Sor*^*tm1(CAG-RABVgp4,-TVA)Arenk*^/J, #024708, Jackson Laboratories], *n* = 18 3–8 month-old homozygous male Chx10-cre mice [Tg(Vsx2-cre)TC9Gsat/Mmucd, MMMRRC 36672], and *n* = 18 8–10 week-old homozygous female Ai14 mice [B6.Cg-*Gt(ROSA)26Sor*^*tm14(CAG-tdTomato)Hze*^/J, #007914, Jackson Laboratories]. A total of *n* = 12 F344 rats (F344/NHsd, Envigo) and *n* = 6 homozygous GFP F344 rats [F344-Tg(UBC-#GFP)F455Rrrc, Rat Resource and Research Center #307] were used for this study. Animals had free access to food and water throughout the study and were group-housed in ventilated cages on a 12-h light/12-h dark cycle (light cycle = 6:00 am–6:00 pm), with ambient temperature between 20–23 °C and 30–70% humidity. During the study, five animals died during surgery or post-operative recovery.

### Spinal cord injury surgeries

All surgeries were performed under deep anesthesia using a combination of ketamine (25 mg/kg), xylazine (5.8 mg/kg), acepromazine (0.25 mg/kg), and inhaled isoflurane (0.5–1%).

#### Dorsal column lesions

We utilized a cervical (C5) dorsal column lesion for graft phenotypic characterization (Figs. 2–5, S2–S4), because this SCI model enables greater ease of animal care and supports graft survival without a stabilizing matrix^12,49^. Mice were deeply anesthetized, and the dorsal skin between the shoulder blades was shaved and scrubbed with betadine and 70% ethanol. The skin and muscle overlying the cervical spinal cord was incised, and a laminectomy was performed at spinal level C4. A tungsten wire knife with an extruded diameter of 1.0–1.5 mm (McHugh Milieux, Downers Grove, IL) was centered above the spinal cord midline, retracted, and inserted to a depth of 0.8 mm below the dorsal spinal cord surface. The arc of the knife was then extruded and raised to transect the spinal cord dorsal columns.

#### Contusions

We utilized a thoracic (T12) contusion as a clinically relevant SCI model with which to assess locomotor recovery (Fig. 6). Mice were deeply anesthetized, and the dorsal aspect of the lower back was shaved and scrubbed with betadine and 70% ethanol. The skin and muscle overlying the thoracic spinal cord was incised and a laminectomy was performed over vertebral level T12. Moderate spinal cord contusion SCI was delivered at T12 using an Infinite Horizon spinal impactor device (IH-0400, Precision Systems and Instrumentation, Fairfax, VA), using a force of 50 kdynes and a 1 s dwell time with a 1.3-mm impactor tip.

### Neural progenitor cell isolation, culture, and transplantation

#### NPC isolation

Mouse embryos were generated through timed mating^49^. Adult female mice received intraperitoneal injections of luteinizing hormone-releasing hormone (5 I.U.; Sigma-Aldrich #L-4513) between 9:00 and 10:00 am. Four days later, a single female mouse was placed into a single male mouse’s home cage between 4:00 and 5:00 pm, and returned to her home cage the following morning between 9:00 and 10:00 am. Embryos were harvested in the morning (between 7:00 and 8:00 am) at 11, 12, or 13 days after separation for E11.5, E12.5, or E13.5 embryos, respectively. The developmental age of the embryos was confirmed visually using morphological landmarks for each embryonic age (Fig. 1b)^30^. Some embryos that appeared underdeveloped compared to littermates were excluded; we observed that this happened more frequently with embryos obtained from Ai14 dams than with other strains. Spinal cords were extracted from the embryos, and meninges were removed. A subset of E12.5 spinal cords were cut with a scalpel in order to separate the dorsal and ventral aspects of the spinal cord^12^. NPCs were isolated from embryonic spinal cord tissue using a previously published protocol^49,99^. First, cords were digested in 0.125% trypsin at 37 °C for 8–12 min. No more than 10 spinal cords were pooled in a single cell preparation. 10 mL of 10% fetal bovine serum in Dulbecco’s Modified Eagle Medium was added to halt the trypsinization reaction, and spinal cords were centrifuged at 600 RCF for 2 min. The supernatant was removed and tissue was gently triturated in 2 mL of Neurobasal Medium + 2% B27 Supplement (NBM/B27) until the cell suspension appeared milky and homogeneous (~15–20 passes through a P1000 pipette tip). Cell suspensions were then centrifuged at 600 RCF for 2 min. The supernatant was removed and cells were resuspended in 2–3 mL of NBM/B27, then passed through a 40-μm cell strainer. Cell viability was assessed by trypan blue exclusion and confirmed to be >95% in all cases. Cells were stored on ice in NBM/B27 until use.

#### NPC transplantation

Cells were resuspended to a concentration of 500,000 viable cells/μL in Hank’s Balanced Salt Solution (HBSS, Gibco) immediately before transplantation. For spinal cord dorsal column lesions, NPCs were transplanted within 5 minutes after SCI. We chose this acute timepoint because we have previously observed that NPC grafts exhibit good survival and integration in this model^12,49,100^, and because combining the SCI and grafting procedures into one surgical session minimizes the number of surgical procedures performed. A volume of 2.0 μL containing 1.0 million cells was injected directly into the lesion cavity at a depth of −0.50 to −0.80 mm via pulled glass micropipette using a PicoSpritzer II (General Valve, Inc., Fairfield, NJ), over a period of 5 min. For spinal cord contusion SCI, cells were transplanted 2 weeks after the SCI; this delay was chosen to minimize the negative effects of inflammation on graft survival. Cells were resuspended in fibrinogen and thrombin components (“fibrin matrix”)^99^. “Vehicle” treated mice received injection of fibrin matrix without cells. A total of 5 μL containing 1.5 million cells was injected around the contusion epicenter, over a total of 6 sites (D/V: −0.80 mm; M/L: +/− 0.35 mm; A/P: +0.25, 0.00, +0.25 mm). For this experiment, subjects were assigned to treatment group based on BMS scores at day 14 post-SCI, such that the inter-group means were not significantly different prior to treatment. Surgeries took place over multiple days, using 1–3 litters of embryos per day to obtain donor cells. Following NPC transplantation, post-operative recovery was performed as described above.

##### NPC culture

A total of 1 million cells per well were plated in 48-well culture plates coated with poly-D-lysine (Gibco). Cells were cultured in 250 μL of NBM/B27 + 1% penicillin-streptomycin-glutamine (Gibco). Cells were maintained at 37 °C/5% CO_2_ for either 24 or 72 h with once-daily media changes. At the end of the experiment, media was removed and cells were fixed with 2% PFA for 20 min at room temperature then washed and stored in PBS at 4 °C. Sets of at least three biological replicates were used for immunostaining and analysis.

### Post-operative care

Following all surgeries, incised muscles were sutured with 4-0 silk sutures, and stainless steel wound clips were used to close the incised skin. Antibiotic powder (Neo-Predef, Zoetis Inc, Kalamazoo, MI) was applied to the sutured muscles prior to skin closure. Post-operative care consisted of subcutaneous injection of banamine (0.05 mg/kg) and ampicillin (0.05 mg/kg) in lactated Ringer’s solution (0.5 mL) once daily for 3 days, and animal cages remained half on/half off heating pads for 72 h post-surgery. Daily health checks were performed for the first week, followed by weekly health checks for the duration of the study. Mice grooming behaviors and weight were monitored on a weekly basis. For mice that received thoracic spinal cord contusions, bladders were manually expressed twice daily (~9:00 am and 5:00 pm) until animals recovered the ability to void bladders on their own.

### Rat transplantation experiment

For the experiment utilizing rats (Fig. S4), E13, E14, and E15 GFP^+^ mouse embryos were generated through timed mating as described above. Spinal cords were extracted from the embryos, and spinal cord NPCs were isolated as described above. Cell viability was assessed by trypan blue exclusion and confirmed to be >95% in all cases. Cells were stored on ice in NBM/B27 until use. Rats received C5 dorsal column lesions using the same technique as above, except for the following changes: a tungsten wire knife with an extruded diameter of 1.5–2.0 mm (McHugh Milieux, Downers Grove, IL) was used, and the wire knife was inserted to a depth of 1.1 mm below the dorsal spinal cord surface prior to transection of the overlying axons. NPCs (total of 1.2 million cells in a volume of 3 μL were transplanted into sites of SCI immediately following the injury, and animals received the same post-operative care as above. Animals were sacrificed 4 weeks after NPC transplantation and transcardially perfused with saline followed by 4% paraformaldehyde, and tissue was collected for immunohistochemical analysis.

### Behavioral testing

All behavioral testing and analysis was performed by experimenters blinded to treatment condition.

#### Open field locomotion

Hindlimb locomotor function was assessed using the Basso Mouse Scale for Locomotion^87^. An open field made of acrylic, measuring ~1 ft × 3 ft, was used for locomotor testing. Prior to testing, mice were first acclimated to the open field environment 30 min daily for 3 days. Baseline scores were collected prior to surgery, and all mice exhibited baseline BMS scores of 9. Following SCI, BMS scores were obtained on 1-, 3-, 5-, and 7 DPI, then once weekly until 70 DPI. At each time point, scores for both hindlimbs were averaged to produce a final score. For analysis, behavior data on 21–70 DPI was represented as change from 14 DPI (immediately prior to treatment).

#### Thermal sensitivity testing

Thermal pain sensation was assessed using the Hargreaves test^101^. We used a Plantar Analgesia Meter with a heated base (Model 390 G, IITC Life Science Inc., Woodland Hills, CA). Prior to testing, mice were first acclimated to the acrylic enclosures placed upon the heated glass surface for 30 min daily for 3 days. During the test period, a light beam with intensity of 30% was delivered to the plantar surface of each hindpaw. The latency of the animal to withdrawal its paw from the light source was automatically recorded by the IITC software, and latency scores were collected for each hindpaw and then averaged. The cutoff time was set to 20 s to avoid tissue damage. Baseline scores were collected prior to surgery. After SCI, scores were collected once weekly from 14 DPI until 70 DPI. On some days, mice did not plantar place sufficiently to obtain reliable scores; data are missing in such instances. For analysis, behavior data on 21–70 DPI was represented as change from 14 DPI (immediately prior to treatment). Mice with missing scores on 14 DPI were excluded from analysis.

### Tissue clearing and lightsheet microscopy

Spinal cords were collected at four weeks post-grafting following transcardial perfusion and overnight post-fixation in 4% paraformaldehyde in PBS. The cervical and upper thoracic regions of the post-fixed spinal cords were dissected and the meninges were removed for iDISCO+ tissue clearing^102,103^. Briefly, spinal cords were dehydrated in a methanol/water gradient (0, 20%, 40%, 60%, 80%, 100%; 1 h each) and kept in 66% dichloromethane (DCM) in methanol overnight. The samples were then washed twice with 100% methanol for 30 min and rehydrated in a methanol/water gradient (80%, 60%, 40%, 20%; 1 h each) followed by a 30 min wash in PBS. The samples were then washed twice with PBS containing 0.2% Triton X-100 and 0.02% sodium azide (PTx.2 solution) for 30 min. To permeabilize and block the spinal cord tissue, samples were incubated overnight at 37 °C in PTx.2 solution containing 20% dimethyl sulfoxide (DMSO), 5% normal donkey serum (NDS), and 0.2% (w/v) glycine. Samples were then incubated for four days at 37 °C in PBS containing 0.2% Tween-20, 0.02% sodium azide, and 0.01% (w/v) heparin (PTwH solution) with the following antibodies: rabbit anti-GFP (1:200) and mouse anti-NeuN (1:200, Clone 1B7). The primary antibody solution was replaced after the first two days of staining. Samples were then washed in PTwH solution five times for 30 min and placed in PTwH solution overnight. Samples were then incubated for four days at 37 °C in PTwH containing the following secondary antibodies: donkey anti-rabbit AlexaFluor Plus 488 (1:200) and donkey anti-mouse AlexaFluor Plus 555 (1:200). The secondary antibody solution was replaced after the first two days of staining. The spinal cords were then washed in PTwH solution five times for 30 min each and placed in PTwH solution overnight. After washing, the spinal cords were embedded in 2% agarose and dehydrated in a methanol/water gradient as described above followed by an overnight incubation in 100% methanol. The spinal cords were washed in 66% DCM in methanol for 3 h, washed twice in DCM for 15 min, and then equilibrated for two days in ethyl cinnamate with gentle rocking. Cleared samples were then imaged on a Zeiss Lightsheet 7 with a 5× objective (0.16 NA). Tiled lightsheet images were stitched and processed in Imaris software (Bitplane). The GFP^+^ graft cells were digitally isolated using the surface function and 3D movies were generated in Imaris.

### Tissue processing and immunohistochemistry

#### Tissue processing

At the completion of the study, animals were euthanized by overdose with anesthetic cocktail and transcardially perfused with 0.1 M phosphate buffer (PB) followed by 4% paraformaldehyde (PFA) in 0.1 M PB. Spinal columns were post-fixed in 4% PFA in 0.1 M PB overnight at 4 °C, then cryopreserved in 30% sucrose in 0.1 M PB for at least 3 days at 4 °C. Spinal cords were removed and kept in 30% sucrose solution at 4 °C until cryosectioning. Tissue blocks were embedded in Tissue-Tek OCT compound (VWR) and frozen on dry ice. Spinal cord tissue was cryosectioned in the sagittal or coronal planes to a thickness of 20 μm. Sections were collected into a 24-well plate and stored at 4 °C.

#### Immunohistochemistry

Either a 1-in-6 or a 1-in-12 tissue series was used for each set of immunohistochemistry. Tissue sections were washed in tris-buffered saline (TBS) three times for 10 min each. For detection of some antigens (calbindin, parvalbumin, Foxp2, Chx10, ChAT, Bhlhb5, Olig2, and Brn3a), tissue sections were incubated in ice-cold 100% methanol for 5 minutes, then washed in TBS again before blocking. For detection of Sox9, biotin amplification was used^104^. Sections were then blocked in tris-buffered saline (TBS) containing 5% donkey serum (Lampire Biological Laboratories, #7332100) and 0.25% Triton-X-100 (Sigma-Aldrich) for 1 h at room temperature. Sections were then incubated with primary antibodies (Supp. Table 1) diluted in blocking solution overnight at 4 °C, except for Tlx3 and Lbx1 primary antibodies, which were incubated at room temperature. The next day, sections were washed in TBS three times for 10 min each, then incubated with AlexaFluor-conjugated secondary antibodies (Supp. Table 1) in blocking solution for 2 h at room temperature. Sections were washed again in TBS three times for 10 min each, with the final wash containing DAPI (5 μg/mL, Sigma Aldrich, D9542). Tissue sections were then mounted onto gelatin-coated slides, rinsed in distilled water, air-dried, and coverslipped with Mowiol mounting medium.

### Immunocytochemistry

Fixed cells were blocked for one hour in 5% donkey serum in TBS. Cells were then incubated with primary antibodies (Supp. Table 1) in blocking solution for 1.5 h at room temperature. Cells were washed in TBS three times for 5 min each, then incubated with AlexaFluor-conjugated secondary antibodies (Supp. Table 1) in blocking solution for 1 h at room temperature. Cells were then incubated in DAPI for 10 min, then washed in TBS three times, 5 min each. Cells were left in the final TBS wash for imaging.

### Image acquisition

Slides were imaged using a Nikon Eclipse upright fluorescent microscope equipped with a Prior Scientific XY motorized stage and a Zyla 4.2 PLUS monochrome camera (Andor). Nikon NIS-Elements software was used for image acquisition and XY stitching. For a given batch of slides that received the same immunolabeling, images were acquired using the same acquisition settings across all samples. To generate representative images (not used for quantification), the Extended Depth of Focus module in NIS-Elements was sometimes used to create focused images from Z-stacks. Images were exported as TIFF files for analysis. Fixed and fluorescently labeled cells were imaged using a Nikon Eclipse Ti2 inverted fluorescent microscope with NIS-Elements software.

### Image analysis

All image analysis was performed in a blinded fashion by two independent experimenters using ImageJ or FIJI software. Images of GFP, tdTomato, and/or NeuN immunoreactivity were used to draw regions of interest (ROIs) around grafts. Automated cell counting methods were always validated by manual counts. Any samples that exhibited poor immunostaining, or which did not contain graft tissue, were excluded from analysis. Cell type-specific markers were always overlaid with the DAPI channel during analysis to ensure that any staining artifacts were not included in quantification.

#### NeuN, Sox9, and Olig2 quantification

Immunoreactivity within graft ROIs was thresholded for each marker using the ImageJ Auto Local Threshold function with Bernsen’s thresholding method^105^. Watershed was applied to binary images and the Analyze Particles function was used to count the total number of NeuN^+^, Sox9^+^, or Olig2^+^ cells.

#### Axon outgrowth quantification

A 1-in-6 series of tissue was used to calculate axon outgrowth. GFP fluorescence was overexposed so that fine GFP^+^ processes were visible at sites distant from the main body of the graft. Graft ROIs were translated in 250-μm increments for 2 mm in rostral and caudal directions throughout the host spinal cord. At each increment, the total number of GFP^+^ axons crossing the leading edge of the ROI was manually counted. The total number of axons in the 1-in-6 tissue series was multiplied by 6 to obtain the extrapolated axon outgrowth for the whole graft, and then this number was divided by the graft volume [(graft area in each ROI) × 6 × section thickness]. Data are represented as the total graft axon outgrowth, normalized to graft volume.

#### Cell subtype quantification

For other cell type-specific markers except for clustered calbindin^+^ neurons, cells were manually counted using the Cell Counter plugin in FIJI. To determine colocalization of two or more markers, image stacks containing multiple image channels were analyzed. For the quantification of clustered calbindin^+^ neurons, ROIs were drawn around the cell clusters to calculate the total area of grafts occupied by these clusters.

#### 5-HT and CGRP axon quantification

Axons were manually traced in FIJI, and the total length of axons within the graft was normalized to graft volume.

### Statistics and reproducibility

Transplantation experiments were repeated using at least two different batches of NPC donor cells per treatment group. A minimum of five mice were used in each experimental group. GraphPad Prism 8 and Jamovi version 2.3 software packages were used to perform statistical analysis. Details for all statistical tests are provided in Supplementary Table 2. All data are presented as mean ± SEM. Statistical significance was defined as *P* < 0.05. All tests were two-tailed.

### Reporting summary

Further information on research design is available in the Nature Portfolio Reporting Summary linked to this article.

## Supplementary information

Dulin_Peer Review File

Supplementary Information

Description of Additional Supplementary Files

Supplementary Data 1

Supplementary Movie 1

Reporting Summary

## Data Availability

All data generated or analyzed during this study are included in this published article, the supplementary files, and the source data. Raw source data for all figures and supplemental figures can be found in Supplementary Data 1. Raw data will be made available upon request, within two weeks of the request. In addition, data from this study are available in a FAIR data repository, the Open Data Commons for Spinal Cord Injury (odc-sci.org), and can be accessed at https://odc-sci.org/data/873.
