# Publisher Correction: Developmental stage of transplanted neural progenitor cells influences anatomical and functional outcomes after spinal cord injury in mice

**DOI:** 10.1038/s42003-023-05018-3

**Published:** 2023-06-13

**Authors:** Miriam Aceves, Ashley Tucker, Joseph Chen, Katie Vo, Joshua Moses, Prakruthi Amar Kumar, Hannah Thomas, Diego Miranda, Gabrielle Dampf, Valerie Dietz, Matthew Chang, Aleena Lukose, Julius Jang, Sneha Nadella, Tucker Gillespie, Christian Trevino, Andrew Buxton, Anna L. Pritchard, Peyton Green, Dylan A. McCreedy, Jennifer N. Dulin

**Affiliations:** 1 Department of Biology, Texas A&M University, College Station, TX 77843 USA; 2 Texas A&M Institute for Neuroscience, Texas A&M University, College Station, TX 77843 USA; 3 Department of Biomedical Engineering, Texas A&M University, College Station, TX 77843 USA; 4 Ganado High School, Ganado, TX 77962 USA

**Keywords:** Spinal cord injury, Neural stem cells

Correction to: *Communications Biology* 10.1038/s42003-023-04893-0, published online 19 May 2023.

In this article the affiliation details for Andrew Buxton were incorrectly given as ‘Ganado High School, Ganado, TX 77962, USA’ but should have been ‘Department of Biomedical Engineering, Texas A&M University, College Station, TX 77843, USA’. The original article has been corrected.

