# Supplementary material for: Developmental stage of transplanted neural progenitor cells influences anatomical and functional outcomes after spinal cord injury in mice
Source: Commun Biol. 2023 May 19;6:544. doi: 10.1038/s42003-023-04893-0 (PMC10199026; doi:10.1038/s42003-023-04893-0)
Supplement: Supplementary file 2 — Supplementary Information [file 42003_2023_4893_MOESM2_ESM.pdf]

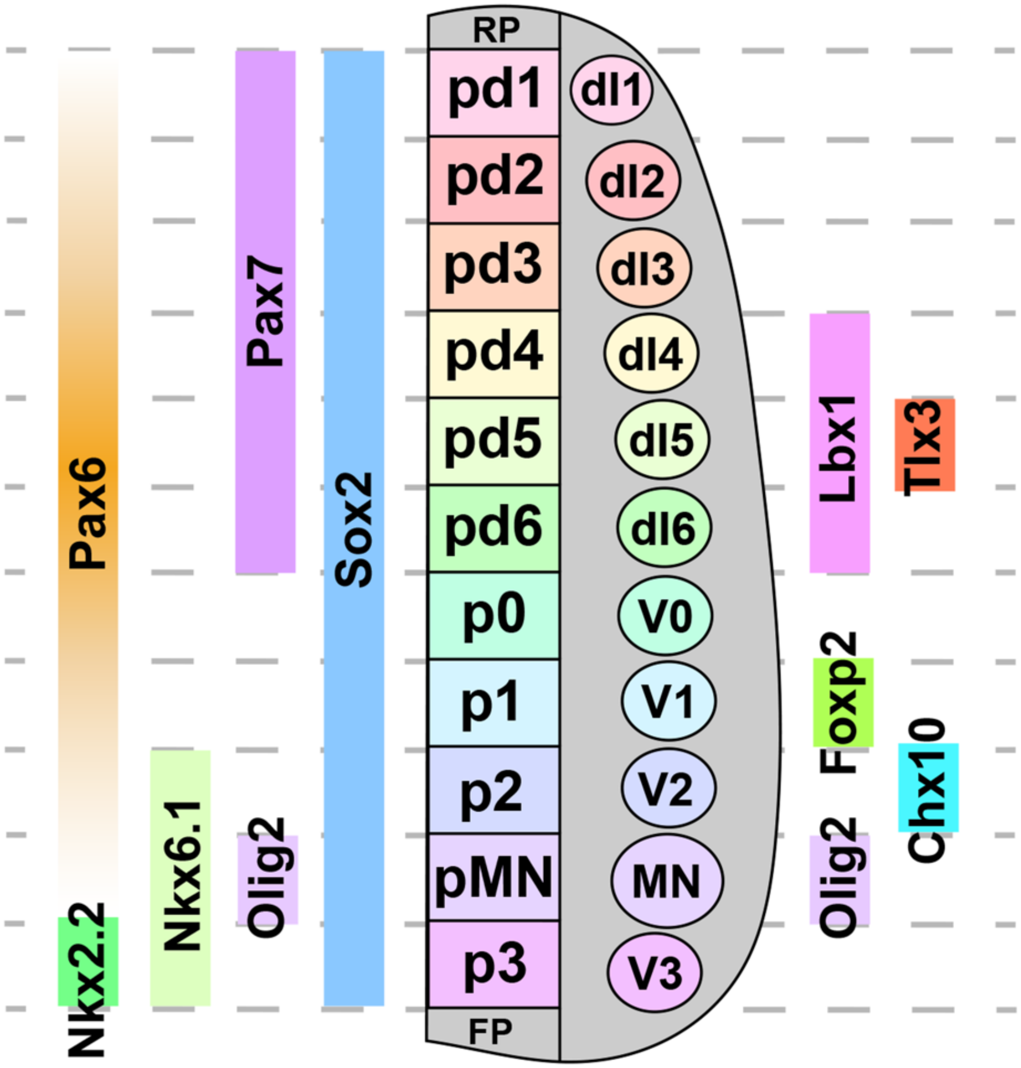

Supplementary Figure 1. Diagram of transcription factors expressed in progenitors and postmitotic neurons in the developing spinal cord. Illustration shows selected transcription factors that are expressed in spinal cord neural tube progenitor domains (left side; pd1 – p3) or in postmitotic neurons (right side; dI1 – V3). Note that Chx10 is only expressed in the V2a subset of V2 interneurons. RP = roof plate, FP = floor plate. This figure is based on an existing body of knowledge about the transcriptional control of spinal cord specification[1-4].

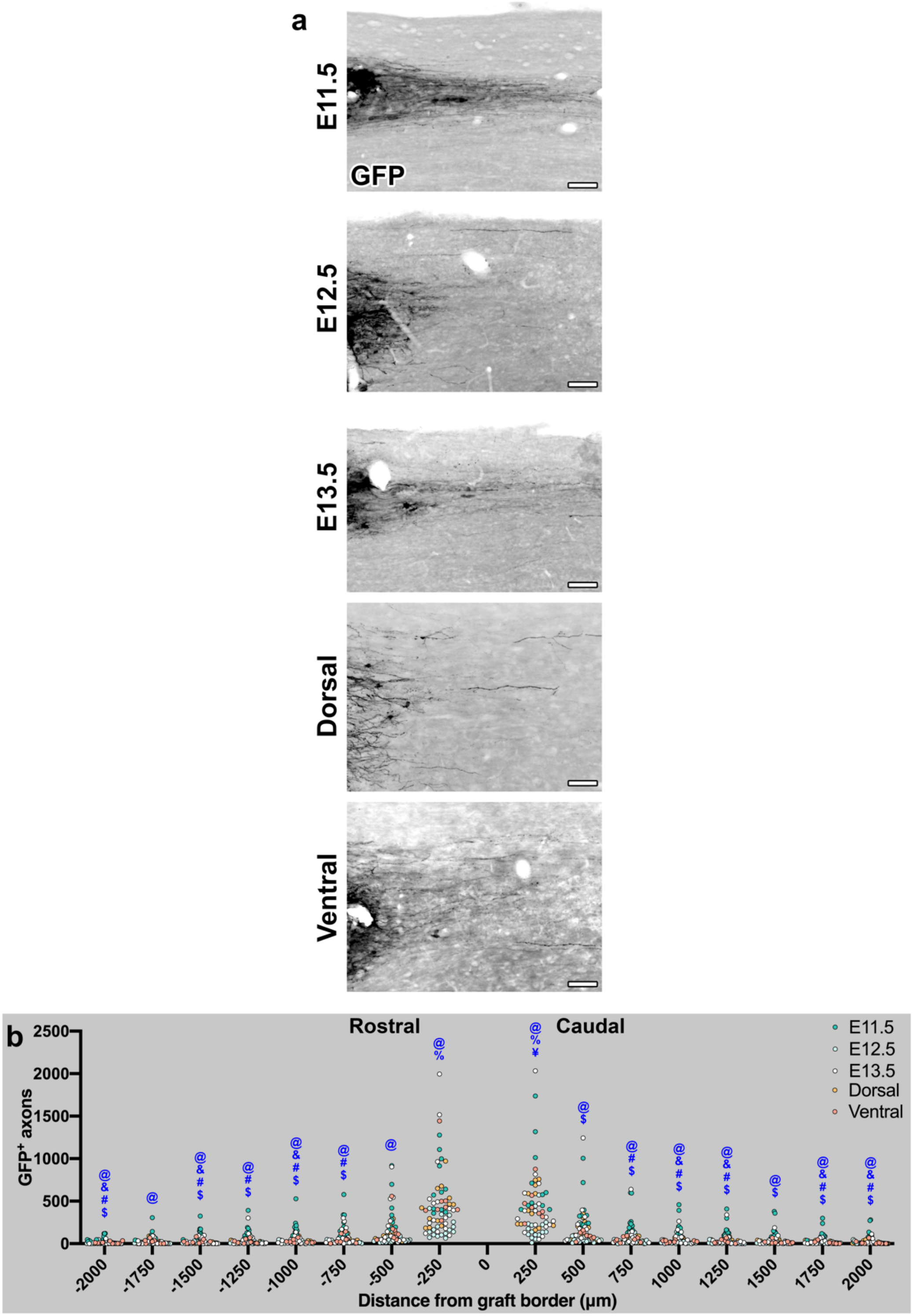

Supplementary Figure 2. Axon outgrowth from GFP^{+} NPC grafts. (**a**) All images are GFP^{+} NPC grafts at 4 weeks post-transplantation into sites of cervical SCI. Scale bars = 100 μm. (**b**) Individual data points for the data in **Figure 2i**.

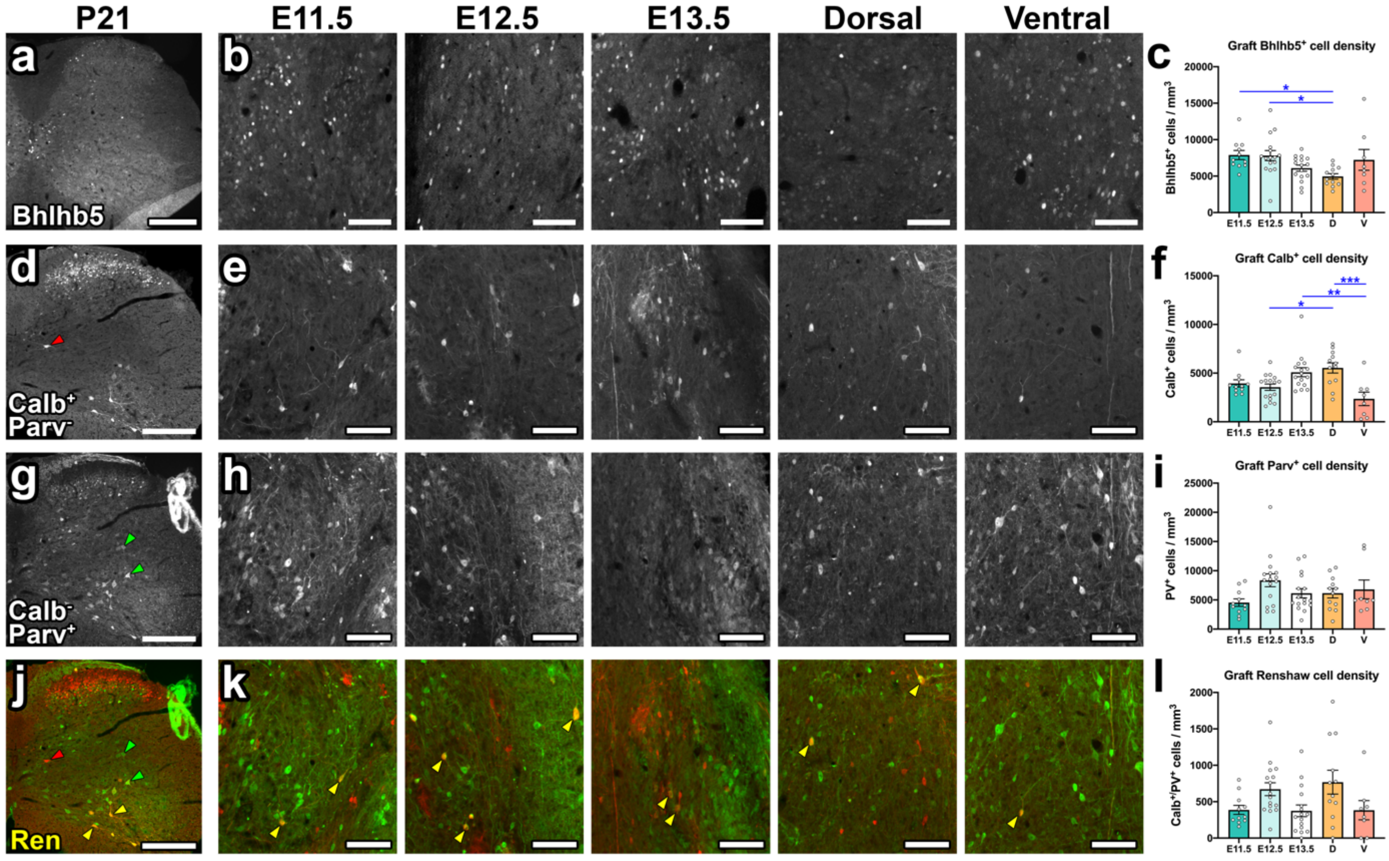

Supplementary Figure 3. Some graft interneuron populations do not vary in abundance with developmental stage of donor NPCs. (**a, d, g, j**) Distribution of cell type-specific markers in intact P21 mouse spinal cord. (**b, e, h, k**) Images of NPC grafts at 4 weeks post-transplantation into sites of cervical SCI. Images within the same column in rows **e**, **h**, and **e** are different fluorescent channels of the same tissue section. (**c, f, i, l**) Quantification of the abundance of (**c**) Bhlhb5$^+$, (**f**) Calb$^+$/Parv$^-$, (**i**) Parv$^+$/Calb$^-$, and (**l**) Calb$^+$/Parv$^+$ Renshaw interneurons in rat NPC grafts. *P<0.05, **P<0.01, ***P<0.001 by one-way ANOVA with Tukey's multiple comparisons test. E11.5 (n=11); E12.5 (n=16); E13.5 (n=16); dorsal (n=12); ventral (n=8). All data are mean ± SEM. Scale bars = 250 µm (**a, d, g, j**); 100 µm (**b, e, h, k**).

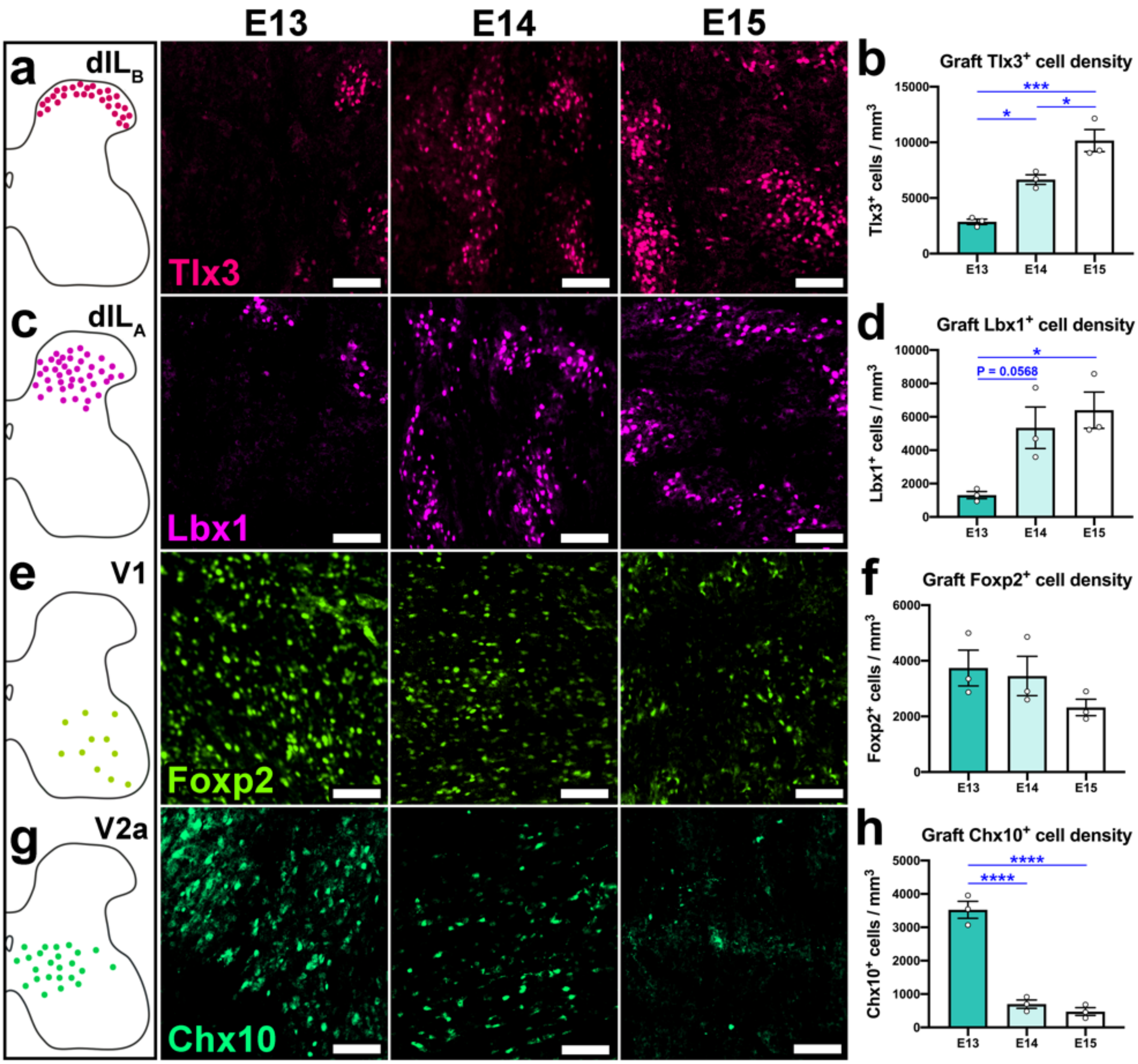

Supplementary Figure 4. Dorsal and ventral spinal cord interneuron populations vary with developmental stage of donor rat neural progenitor cells. (**a, c, e, g**) Images of cell type-specific markers in rat NPC grafts at 4 weeks following transplantation into the injured adult rat cervical spinal cord. Cartoons represent the spatial distribution of each neuronal cell type in the postnatal rat spinal cord. (**b, d, f, h**) Quantification of the abundance of (**b**) Tlx3$^+$, (**d**) Lbx1$^+$, (**f**) Foxp2$^+$, and (**h**) Chx10$^+$ interneurons in rat NPC grafts. *P<0.05, ***P<0.001, ****P<0.0001 by one-way ANOVA with Tukey's multiple comparisons test. E13 (n=3 mice), E14 (n=3 mice), E15 (n=3 mice). All data are mean ± SEM. Scale bars = 100 μm**.**

Supplementary Table 1. Primary and secondary antibodies used in this study.

Antibody	Catalog #	RRID	Dilution
**Primary antibodies**			
Rabbit anti-5-HT	ImmunoStar #20080	AB_572263	1:20,000
Rabbit anti-Bhlhb5	Gift of Dr. Sarah Ross	Not listed	1:1,000
Mouse anti-Brn3a	Millipore #MAB1585	AB_94166	1:200
Chicken anti-Calbindin	EnCor #CPCA-Calb	AB_2572237	1:200
Goat anti-CGRP	Abcam #ab36001	AB_725807	1:1,000
Goat anti-ChAT	Genetex #GTX82725	AB_11162364	1:200
Sheep anti-Chx10	Abcam #ab16141	AB_302278	1:200
Rabbit anti-Foxp2	Abcam #ab16046	AB_2107107	1:200
Chicken anti-GFP	Aves #GFP-1020	AB_10000240	1:1,500
Goat anti-GFP	Rockland #600-101-215	AB_218182	1:1,500
Rabbit anti-GFP	Thermo Fisher Scientific #A-11122	AB_221569	1:200
Guinea pig anti-Lbx1	Gift of Drs. Carmen Birchmeier & Thomas Müller	AB_2532144	1:20,000
Goat anti-mCherry	Sicgen #AB0040-200	AB_2333093	1:1,000
Guinea pig anti-NeuN	Millipore #ABN90	AB_11205592	1:2,000
Mouse anti-NeuN	Biolegend #834501	AB_2564991	1:200
Rabbit anti-Nfib	Sigma #HPA 003956	AB_1854424	1:1,600
Mouse anti-Nkx2.2	DSHB #74.5A5	AB_531794	1:50
Mouse anti-Nkx6.1	DSHB #F55A12	AB_532379	1:50
Rabbit anti-Olig2	Millipore #AB9610	AB_570666	1:200
Rabbit anti-Parvalbumin	Swant #PV27	AB_2631173	1:200
Rabbit anti-Pax6	Biolegend #901301	AB_2565003	1:50
Mouse anti-Pax7	DSHB #Pax7	AB_528428	1:50
Rabbit anti-Sox2	Abcam #ab97959	AB_2341193	1:200
Goat anti-Sox9	R&D Systems #AF3075	AB_2194160	1:200
Rabbit anti-Sox9	Abcam #ab185966	AB_2728660	1:2,000
Rabbit anti-Tlx3	Gift of Drs. Carmen Birchmeier & Thomas Müller	AB_2532145	1:10,000
Rabbit anti-Zfhx4	Sigma #HPA 023837	AB_1859013	1:100
**Secondary antibodies**			
AlexaFluor 488 Affinipure donkey anti-chicken IgY	Jackson ImmunoResearch #703-545-155	AB_2340375	1:1,000
Cy3 Affinipure donkey anti-chicken IgY	Jackson ImmunoResearch #703-165-155	AB_2340363	1:1,000
AlexaFluor 647 Affinipure donkey anti-chicken IgY	Jackson ImmunoResearch #703-605-155	AB_2340379	1:1,000
AlexaFluor 488 Affinipure donkey anti-goat IgG	Jackson ImmunoResearch #705-545-147	AB_2336933	1:1,000
Cy3 Affinipure donkey anti-goat IgG	Jackson ImmunoResearch #705-165-147	AB_2307351	1:1,000
AlexaFluor 647 Affinipure donkey anti-goat IgG	Jackson ImmunoResearch #705-605-147	AB_2340437	1:1,000

AlexaFluor 488 Affinipure donkey anti-guinea pig IgG	Jackson ImmunoResearch #706-545-148	AB_2340472	1:1,000
AlexaFluor 647 Affinipure donkey anti-guinea pig IgG	Jackson ImmunoResearch #706-605-148	AB_2340476	1:1,000
AlexaFluor 488 Affinipure donkey anti-mouse IgG	Jackson ImmunoResearch #715-545-150	AB_2340846	1:1,000
Cy3 Affinipure donkey anti-mouse IgG	Jackson ImmunoResearch #715-165-150	AB_2340813	1:1,000
Alexa Fluor Plus 555 donkey anti-mouse IgG	Thermo Fisher Scientific #A32773	AB_2762848	1:200
AlexaFluor 647 Affinipure donkey anti-mouse IgG	Jackson ImmunoResearch #715-605-150	AB_2340862	1:1,000
AlexaFluor 488 Affinipure donkey anti-rabbit IgG	Jackson ImmunoResearch #711-545-152	AB_2313584	1:1,000
Alexa Fluor Plus 488 donkey anti-rabbit IgG	Thermo Fisher Scientific #A32790	AB_2762833	1:200
Cy3 Affinipure donkey anti-rabbit IgG	Jackson ImmunoResearch #711-165-152	AB_2307443	1:1,000
AlexaFluor 647 Affinipure donkey anti-rabbit IgG	Jackson ImmunoResearch #711-605-152	AB_2492288	1:1,000
Cy3 Affinipure donkey anti-sheep IgG	Jackson ImmunoResearch #713-165-003	AB_2340727	1:1,000
AlexaFluor Affinipure 647 donkey anti-sheep IgG	Jackson ImmunoResearch #713-605-003	AB_2340750	1:1,000

Supplementary Table 2. Detailed description of statistical analyses used in this study.

Fig.	Parameter	Group size (n)	Statistical test	Significance level
1c	Percent $Sox2^{+}$ cells (24 h)	E11.5 (n=4 wells); E12.5 (n=5 wells); E13.5 (n=7 wells)	Ordinary one-way ANOVA with Tukey's multiple comparisons test	F (2, 13) = 4.883; P = 0.0262<br><br>E11.5 vs. E13.5: P = 0.0227
1d	Percent $Olig2^{+}$ cells (24 h)	E11.5 (n=4 wells); E12.5 (n=6 wells); E13.5 (n=7 wells)	Ordinary one-way ANOVA with Tukey's multiple comparisons test	F (2, 14) = 4.012; P = 0.0419<br><br>No significant differences among groups
1e	Percent $Pax6^{+}/Pax7^{-}$ cells (24 h)	E11.5 (n=4 wells); E12.5 (n=6 wells); E13.5 (n=6 wells)	Ordinary one-way ANOVA with Tukey's multiple comparisons test	F (2, 13) = 122.2; P < 0.0001<br><br>E11.5 vs. E12.5: P < 0.0001<br>E11.5 vs. E13.5: P < 0.0001
1f	Percent $Pax7^{+}$ cells (24 h)	E11.5 (n=4 wells); E12.5 (n=6 wells); E13.5 (n=6 wells)	Ordinary one-way ANOVA with Tukey's multiple comparisons test	F (2, 13) = 0.1213; P = 0.8868<br><br>No significant differences among groups
1g	Percent $Nkx2.2^{+}$ cells (24 h)	E11.5 (n=4 wells); E12.5 (n=6 wells); E13.5 (n=6 wells)	Ordinary one-way ANOVA with Tukey's multiple comparisons test	F (2, 13) = 4.124; P = 0.0410<br><br>E11.5 vs. E13.5: P = 0.0364
1h	Percent $Nkx6.1^{+}$ cells (24 h)	E11.5 (n=4 wells); E12.5 (n=6 wells); E13.5 (n=7 wells)	Ordinary one-way ANOVA with Tukey's multiple comparisons test	F (2, 14) = 3.127; P = 0.0754<br><br>No significant differences among groups
1i	Percent $NeuN^{+}$ cells (72 h)	E11.5 (n=4 wells); E12.5 (n=9 wells); E13.5 (n=6 wells)	Ordinary one-way ANOVA with Tukey's multiple comparisons test	F (2, 16) = 0.1832; P = 0.8343<br><br>No significant differences among groups
1j	Percent $Sox9^{+}$ cells (72 h)	E11.5 (n=4 wells); E12.5 (n=6 wells); E13.5 (n=5 wells)	Ordinary one-way ANOVA with Tukey's multiple comparisons test	F (2, 12) = 3.184; P 0.0778<br><br>No significant differences among groups
1k	Percent $Olig2^{+}$ cells (72 h)	E11.5 (n=4 wells); E12.5 (n=9 wells); E13.5 (n=6 wells)	Ordinary one-way ANOVA with Tukey's multiple comparisons test	F (2, 16) = 0.9079; P = 0.4232<br><br>No significant differences among groups
1l	Percent $Sox2^{+}$ cells (72 h)	E11.5 (n=4 wells); E12.5 (n=6 wells); E13.5 (n=7 wells)	Ordinary one-way ANOVA with Tukey's multiple comparisons test	F (2, 14) = 0.2965; P = 0.7479<br><br>No significant differences among groups
1m	Percent $Lbx1^{+}$ cells (72 h)	E11.5 (n=4 wells); E12.5	Ordinary one-way ANOVA with	F (2, 14) = 7.669; P = 0.0056

		(n=6 wells); E13.5 (n=7 wells)	Tukey's multiple comparisons test	E11.5 vs. E13.5: P = 0.0394<br>E12.5 vs. E13.5: P = 0.0066
1n	Percent Tlx3$^+$ cells (72 h)	E11.5 (n=4 wells); E12.5 (n=6 wells); E13.5 (n=5 wells)	Ordinary one-way ANOVA with Tukey's multiple comparisons test	F (2, 12) = 3.014; P = 0.0870<br><br>No significant differences among groups
1o	Percent Chx10$^+$ cells (72 h)	E11.5 (n=4 wells); E12.5 (n=6 wells); E13.5 (n=7 wells)	Ordinary one-way ANOVA with Tukey's multiple comparisons test	F (2, 14) = 37.39; P < 0.0001<br><br>E11.5 vs. E12.5: P < 0.0001<br>E11.5 vs. E13.5: P < 0.0001
1p	Percent Foxp2$^+$ cells (72 h)	E11.5 (n=4 wells); E12.5 (n=6 wells); E13.5 (n=7 wells)	Ordinary one-way ANOVA with Tukey's multiple comparisons test	F (2, 14) = 15.44; P = 0.0003<br><br>E11.5 vs. E12.5: P = 0.0025<br>E11.5 vs. E13.5: P = 0.0002
2d	Graft neuron density	E11.5 (n=11); E12.5 (n=16); E13.5 (n=16); dorsal (n=12); ventral (n=8)	Ordinary one-way ANOVA with Tukey's multiple comparisons test	F (4, 58) = 9.818; P < 0.0001<br><br>E11.5 vs. E12.5: P = 0.0039<br>E11.5 vs. dorsal: P < 0.0001<br>E11.5 vs. ventral: P = 0.0068<br>E13.5 vs. dorsal: P = 0.0009
2f	Graft astrocyte density	E11.5 (n=11); E12.5 (n=16); E13.5 (n=16); dorsal (n=12); ventral (n=8)	Ordinary one-way ANOVA with Tukey's multiple comparisons test	F (4, 58) = 5.214; P = 0.0012<br><br>E11.5 vs. ventral: P = 0.0370<br>E12.5 vs. dorsal: P = 0.0156<br>E12.5 vs. ventral: P = 0.0020
2h	Graft oligodendrocyte density	E11.5 (n=11); E12.5 (n=16); E13.5 (n=15); dorsal (n=12); ventral (n=8)	Ordinary one-way ANOVA with Tukey's multiple comparisons test	F (4, 57) = 2.974; P = 0.0267<br><br>E12.5 vs. ventral: P = 0.0322
2i	Graft axon outgrowth	E11.5 (n=11); E12.5 (n=16); E13.5 (n=16); dorsal (n=12); ventral (n=8)	Two-way repeated measures ANOVA with Tukey's multiple comparisons test	Distance x Treatment main effect: F (60, 870) = 3.242, P < 0.0001<br>Distance main effect: F (1.393, 80.79) = 88.77, P < 0.0001<br>Treatment main effect: F (4, 58) = 8.305, P < 0.0001<br>Subject main effect: F (58, 870) = 10.85, P < 0.0001<br><br>-2000 µm:<br>E11.5 vs. E12.5: P = 0.0009<br>E11.5 vs. E13.5: P = 0.0010<br>E11.5 vs. dorsal: P = 0.0009<br>E11.5 vs. ventral: P = 0.0020<br><br>-1750 µm:<br>E11.5 vs. E12.5: P = 0.0217<br><br>-1500 µm:<br>E11.5 vs. E12.5: P = 0.0027<br>E11.5 vs. E13.5: P = 0.0079

				E11.5 vs. dorsal: P = 0.0039<br>E11.5 vs. ventral: P = 0.0092<br><br>-1250 µm:<br>E11.5 vs. E12.5: P = 0.0075<br>E11.5 vs. dorsal: P = 0.0132<br>E11.5 vs. ventral: P = 0.0154<br><br>-1000 µm:<br>E11.5 vs. E12.5: P = 0.0082<br>E11.5 vs. E13.5: P = 0.0161<br>E11.5 vs. dorsal: P = 0.0149<br>E11.5 vs. ventral: P = 0.0112<br><br>-750 µm:<br>E11.5 vs. E12.5: P = 0.0070<br>E11.5 vs. dorsal: P = 0.0192<br>E11.5 vs. ventral: P = 0.0180<br><br>-500 µm:<br>E11.5 vs. E12.5: P = 0.0167<br><br>-250 µm:<br>E11.5 vs. E12.5: P = 0.0024<br>E12.5 vs. dorsal: P = 0.0213<br><br>+250 µm:<br>E11.5 vs. E12.5: P = 0.0121<br>E12.5 vs. dorsal: P = 0.0386<br>E12.5 vs. ventral: P = 0.0391<br><br>+500 µm:<br>E11.5 vs. E12.5: P = 0.0116<br>E11.5 vs. ventral: P = 0.0247<br><br>+750 µm:<br>E11.5 vs. E12.5: P = 0.0055<br>E11.5 vs. dorsal: P = 0.221<br>E11.5 vs. ventral: P = 0.0105<br><br>+1000 µm:<br>E11.5 vs. E12.5: P = 0.0048<br>E11.5 vs. E13.5: P = 0.0290<br>E11.5 vs. dorsal: P = 0.0117<br>E11.5 vs. ventral: P = 0.0059<br><br>+1250 µm:<br>E11.5 vs. E12.5: P = 0.0041<br>E11.5 vs. E13.5: P = 0.0291<br>E11.5 vs. dorsal: P = 0.0086<br>E11.5 vs. ventral: P = 0.0056<br><br>+1500 µm:<br>E11.5 vs. E12.5: P = 0.0282<br>E11.5 vs. ventral: P = 0.0317

				+1750 µm:<br>E11.5 vs. E12.5: $P = 0.0091$<br>E11.5 vs. E13.5: $P = 0.0135$<br>E11.5 vs. dorsal: $P = 0.0152$<br>E11.5 vs. ventral: $P = 0.0082$<br><br>+2000 µm:<br>E11.5 vs. E12.5: $P = 0.0046$<br>E11.5 vs. E13.5: $P = 0.0093$<br>E11.5 vs. dorsal: $P = 0.0093$<br>E11.5 vs. ventral: $P = 0.0074$
3c	Graft $Tlx3^+$ cell density	E11.5 (n=11);<br>E12.5 (n=16);<br>E13.5 (n=16);<br>dorsal (n=12);<br>ventral (n=8)	Ordinary one-way ANOVA with Tukey's multiple comparisons test	$F(4, 58) = 18.27$; $P < 0.0001$<br><br>E11.5 vs. E12.5: $P = 0.0211$<br>E11.5 vs. E13.5: $P < 0.0001$<br>E11.5 vs. dorsal: $P = 0.0071$<br>E12.5 vs. E13.5: $P = 0.0048$<br>E12.5 vs. ventral: $P = 0.0004$<br>E13.5 vs. ventral: $P < 0.0001$<br>Dorsal vs. ventral: $P = 0.0001$
3f	Graft $Lbx1^+$ cell density	E11.5 (n=11);<br>E12.5 (n=16);<br>E13.5 (n=16);<br>dorsal (n=12);<br>ventral (n=8)	Ordinary one-way ANOVA with Tukey's multiple comparisons test	$F(4, 58) = 27.24$; $P < 0.0001$<br><br>E11.5 vs. E12.5: $P = 0.0027$<br>E11.5 vs. E13.5: $P < 0.0001$<br>E11.5 vs. dorsal: $P = 0.0152$<br>E12.5 vs. E13.5: $P < 0.0001$<br>E12.5 vs. ventral: $P = 0.0001$<br>E13.5 vs. dorsal: $P < 0.0001$<br>E13.5 vs. ventral: $P < 0.0001$<br>Dorsal vs. ventral: $P = 0.0008$
3i	Percent graft $calbindin^+$ cell cluster area	E11.5 (n=11);<br>E12.5 (n=16);<br>E13.5 (n=16);<br>dorsal (n=12);<br>ventral (n=8)	Ordinary one-way ANOVA with Tukey's multiple comparisons test	$F(4, 58) = 22.25$; $P < 0.0001$<br><br>E11.5 vs. E12.5: $P = 0.0063$<br>E11.5 vs. E13.5: $P < 0.0001$<br>E11.5 vs. dorsal: $P = 0.0021$<br>E12.5 vs. E13.5: $P = 0.0011$<br>E12.5 vs. ventral: $P = 0.0001$<br>E13.5 vs. dorsal: $P = 0.0191$<br>E13.5 vs. ventral: $P < 0.0001$<br>Dorsal vs. ventral: $P < 0.0001$
3l	Graft $Brn3a^+$ cell density	E11.5 (n=11);<br>E12.5 (n=16);<br>E13.5 (n=15);<br>dorsal (n=12);<br>ventral (n=8)	Ordinary one-way ANOVA with Tukey's multiple comparisons test	$F(4, 57) = 5.686$; $P = 0.0006$<br><br>E12.5 vs. ventral: $P = 0.0011$<br>Dorsal vs. ventral: $P = 0.0010$
3o	Graft $Foxp2^+$ cell density	E11.5 (n=11);<br>E12.5 (n=16);<br>E13.5 (n=16);<br>dorsal (n=12);<br>ventral (n=8)	Ordinary one-way ANOVA with Tukey's multiple comparisons test	$F(4, 58) = 8.134$; $P < 0.0001$<br><br>E12.5 vs. E13.5: $P = 0.0004$<br>E12.5 vs. dorsal: $P = 0.0001$<br>Dorsal vs. ventral: $P = 0.0311$
3r	Graft $Chx10^+$ cell density	E11.5 (n=11);<br>E12.5 (n=16);<br>E13.5 (n=16);<br>dorsal (n=12);<br>ventral (n=8)	Ordinary one-way ANOVA with Tukey's multiple comparisons test	$F(4, 58) = 4.083$; $P = 0.0055$<br><br>E13.5 vs. ventral: $P = 0.0434$<br>Dorsal vs. ventral: $P = 0.0033$

4c	Chx10-cre::Ai14 graft V2a neuron density	E11.5 (n=7); E12.5 (n=6); E13.5 (n=8)	Ordinary one-way ANOVA with Tukey's multiple comparisons test	F (2, 18) = 7.243; P = 0.0049<br><br>E11.5 vs. E12.5: P = 0.0155;<br>E11.5 vs. E13.5: P = 0.0078
4e	Chx10-cre::Ai14 graft Nfib$^+$ cell density	E11.5 (n=7); E12.5 (n=5); E13.5 (n=8)	Ordinary one-way ANOVA with Tukey's multiple comparisons test	F (2, 17) = 15.01; P = 0.0002<br><br>E11.5 vs. E13.5: P = 0.0001<br>E12.5 vs. E13.5: P = 0.0340
4f	Chx10-cre::Ai14 graft tdTomato$^+$/ Nfib$^+$ cell density	E11.5 (n=7); E12.5 (n=5); E13.5 (n=8)	Ordinary one-way ANOVA with Tukey's multiple comparisons test	F (2, 17) = 0.005486; P = 0.9945<br><br>No significant differences among groups
4h	Chx10-cre::Ai14 graft Zfhx4$^+$ cell density	E11.5 (n=7); E12.5 (n=4); E13.5 (n=7)	Ordinary one-way ANOVA with Tukey's multiple comparisons test	F (2, 15) = 15.19; P = 0.0002<br><br>E11.5 vs. E12.5: P = 0.0230<br>E11.5 vs. E13.5: P = 0.0002
4i	Chx10-cre::Ai14 graft tdTomato$^+$/ Zfhx4$^+$ cell density	E11.5 (n=7); E12.5 (n=4); E13.5 (n=7)	Ordinary one-way ANOVA with Tukey's multiple comparisons test	F (2, 15) = 6.234; P = 0.0107<br><br>E11.5 vs. E13.5: P = 0.0105
5c	Regeneration of 5-HT$^+$ axons in grafts	E11.5 (n=11); E12.5 (n=16); E13.5 (n=16); dorsal (n=12); ventral (n=8)	Ordinary one-way ANOVA with Tukey's multiple comparisons test	F (4, 58) = 11.11; P < 0.0001<br><br>E11.5 vs. E12.5: P < 0.0001<br>E11.5 vs. E13.5: P = 0.0002<br>E11.5 vs. dorsal: P < 0.0001
5d	Regeneration of CGRP$^+$ axons in grafts	E11.5 (n=11); E12.5 (n=16); E13.5 (n=16); dorsal (n=12); ventral (n=8)	Ordinary one-way ANOVA with Tukey's multiple comparisons test	F (4, 58) = 4.277, P = 0.0042<br><br>E11.5 vs. E13.5: P = 0.0380<br>E12.5 vs. E13.5: P = 0.0053<br>E13.5 vs. ventral: P = 0.0475
6b	Contusion displacement scores	Vehicle (n=10); E11.5 (n=10); E12.5 (n=10), E13.5 (n=7)	Ordinary one-way ANOVA with Tukey's multiple comparisons test	F (3, 33) = 1.410, P = 0.2574<br><br>No significant differences among groups
6c	Graft volume	Vehicle (n=10); E11.5 (n=10); E12.5 (n=10), E13.5 (n=7)	Ordinary one-way ANOVA with Tukey's multiple comparisons test	F (2, 24) = 1.255, P = 0.3032<br><br>No significant differences among groups
6d	Graft neuronal density	Vehicle (n=10); E11.5 (n=10); E12.5 (n=10), E13.5 (n=7)	Ordinary one-way ANOVA with Tukey's multiple comparisons test	F (2, 24) = 0.3020, P = 0.7421<br><br>No significant differences among groups
6e	Basso Mouse Scale open field locomotor scores	Vehicle (n=10); E11.5 (n=10); E12.5 (n=10), E13.5 (n=7)	Two-way repeated measures ANOVA	Time x Treatment main effect: F (21, 231) = 1.4884, P = 0.051
6f	Hargreaves scores	Vehicle (n=10); E11.5 (n=8); E12.5 (n=10), E13.5 (n=5)	Repeated measures ANOVA with animals missing scores on day 14 deleted (for all 4 groups and for	All four groups:<br>Time x Treatment main effect: F (21, 203) = 2.599, P = 0.0004<br><br>Three transplanted groups:<br>Time x Treatment main effect: F (14, 126) = 2.382, P = 0.0056

			the 3 transplanted groups only)	
S3c	Graft Bhlhb5$^+$ cell density	E11.5 (n=11); E12.5 (n=16); E13.5 (n=16); dorsal (n=12); ventral (n=8)	Ordinary one-way ANOVA with Tukey's multiple comparisons test	F (4, 58) = 3.485; P = 0.0128<br><br>E11.5 vs. dorsal: P = 0.0365<br>E12.5 vs. dorsal: P = 0.0224
S3f	Graft Calb$^+$/Parv$^-$ cell density	E11.5 (n=11); E12.5 (n=16); E13.5 (n=16); dorsal (n=12); ventral (n=8)	Ordinary one-way ANOVA with Tukey's multiple comparisons test	F (4, 58) = 6.374; P = 0.0003<br><br>E12.5 vs. dorsal: P = 0.0203<br>E13.5 vs. ventral: P = 0.0027<br>Dorsal vs. ventral: P = 0.0007
S3i	Graft Calb$^-$/Parv$^+$ cell density	E11.5 (n=11); E12.5 (n=16); E13.5 (n=16); dorsal (n=12); ventral (n=8)	Ordinary one-way ANOVA with Tukey's multiple comparisons test	F (4, 58) = 2.045; P = 0.0999<br><br>No significant differences among groups
S3l	Graft Renshaw cell density	E11.5 (n=11); E12.5 (n=16); E13.5 (n=16); dorsal (n=12); ventral (n=8)	Ordinary one-way ANOVA with Tukey's multiple comparisons test	F (4, 58) = 3.082; P = 0.0227<br><br>No significant differences among groups
S4b	Graft Tlx3$^+$ cell density	E13 (n=3); E14 (n=3); E15 (n=3)	Ordinary one-way ANOVA with Tukey's multiple comparisons test	F (2, 6) = 32.52; P = 0.0006<br><br>E13 vs. E14: P = 0.0134<br>E13 vs. E15: P = 0.0005<br>E14 vs. E15: P = 0.0195
S4d	Graft Lbx1$^+$ cell density	E13 (n=3); E14 (n=3); E15 (n=3)	Ordinary one-way ANOVA with Tukey's multiple comparisons test	F (2, 6) = 7.792; P = 0.0215<br><br>E13 vs. E15: P = 0.0225
S4f	Graft Foxp2$^+$ cell density	E13 (n=3); E14 (n=3); E15 (n=3)	Ordinary one-way ANOVA with Tukey's multiple comparisons test	F (2, 6) = 1.679; P = 0.2635<br><br>No significant differences among groups
S4h	Graft Chx10$^+$ cell density	E13 (n=3); E14 (n=3); E15 (n=3)	Ordinary one-way ANOVA with Tukey's multiple comparisons test	F (2, 6) = 91.63; P < 0.0001<br><br>E13 vs. E14: P < 0.0001<br>E13 vs. E15: P < 0.0001

Supplementary References

1 Lu, D. C., Niu, T. & Alaynick, W. A. Molecular and cellular development of spinal cord locomotor circuitry. *Frontiers in molecular neuroscience* **8**, 25, doi:10.3389/fnmol.2015.00025 (2015).

2 Alaynick, W. A., Jessell, T. M. & Pfaff, S. L. SnapShot: spinal cord development. *Cell* **146**, 178-178 e171, doi:10.1016/j.cell.2011.06.038 (2011).

3 Lai, H. C., Seal, R. P. & Johnson, J. E. Making sense out of spinal cord somatosensory development. *Development* **143**, 3434-3448, doi:10.1242/dev.139592 (2016).

4 Helms, A. W. & Johnson, J. E. Specification of dorsal spinal cord interneurons. *Curr Opin Neurobiol* **13**, 42-49 (2003).
