# Supplementary material for: Developmental stage of transplanted neural progenitor cells influences anatomical and functional outcomes after spinal cord injury in mice
Source: Commun Biol. 2023 May 19;6:544. doi: 10.1038/s42003-023-04893-0 (PMC10199026; doi:10.1038/s42003-023-04893-0)
Supplement: Supplementary file 3 — Description of Additional Supplementary Files [file 42003_2023_4893_MOESM3_ESM.pdf]

Description of Additional Supplementary Files

File name: Supplementary Movie 1

Description: 3D lightsheet image of GFP+ cells (green) and GFP+/NeuN+ cells (red) in the cleared cervical spinal cord at 4 weeks post-grafting.

File name: Supplementary Data 1

Description: Raw source data for all figures and supplemental figures.
