# Supplementary material for: Developmental stage of transplanted neural progenitor cells influences anatomical and functional outcomes after spinal cord injury in mice
Source: Commun Biol. 2023 May 19;6:544. doi: 10.1038/s42003-023-04893-0 (PMC10199026; doi:10.1038/s42003-023-04893-0)
Supplement: Supplementary file 6 — Reporting Summary [file 42003_2023_4893_MOESM6_ESM.pdf]

nature portfolio

Corresponding author(s): Jennifer Dulin

Last updated by author(s): Apr 25, 2023

Reporting Summary

Nature Portfolio wishes to improve the reproducibility of the work that we publish. This form provides structure for consistency and transparency in reporting. For further information on Nature Portfolio policies, see our Editorial Policies and the Editorial Policy Checklist.

Statistics

For all statistical analyses, confirm that the following items are present in the figure legend, table legend, main text, or Methods section.

n/a	Confirmed	
☐	☒	The exact sample size (n) for each experimental group/condition, given as a discrete number and unit of measurement
☐	☒	A statement on whether measurements were taken from distinct samples or whether the same sample was measured repeatedly
☐	☒	The statistical test(s) used AND whether they are one- or two-sided<br>*Only common tests should be described solely by name; describe more complex techniques in the Methods section.*
☐	☒	A description of all covariates tested
☐	☒	A description of any assumptions or corrections, such as tests of normality and adjustment for multiple comparisons
☐	☒	A full description of the statistical parameters including central tendency (e.g. means) or other basic estimates (e.g. regression coefficient) AND variation (e.g. standard deviation) or associated estimates of uncertainty (e.g. confidence intervals)
☐	☒	For null hypothesis testing, the test statistic (e.g. F, t, r) with confidence intervals, effect sizes, degrees of freedom and P value noted<br>*Give P values as exact values whenever suitable.*
☒	☐	For Bayesian analysis, information on the choice of priors and Markov chain Monte Carlo settings
☒	☐	For hierarchical and complex designs, identification of the appropriate level for tests and full reporting of outcomes
☒	☐	Estimates of effect sizes (e.g. Cohen's d, Pearson's r), indicating how they were calculated

Our web collection on statistics for biologists contains articles on many of the points above.

Software and code

Policy information about availability of computer code

Data collection	Nikon NIS Elements software (AR 5.02.00) was used for image acquisition.
Data analysis	ImageJ 1.53h was used for data analysis.

For manuscripts utilizing custom algorithms or software that are central to the research but not yet described in published literature, software must be made available to editors and reviewers. We strongly encourage code deposition in a community repository (e.g. GitHub). See the Nature Portfolio guidelines for submitting code & software for further information.

Data

Policy information about availability of data

All manuscripts must include a data availability statement. This statement should provide the following information, where applicable:

- Accession codes, unique identifiers, or web links for publicly available datasets
- A description of any restrictions on data availability
- For clinical datasets or third party data, please ensure that the statement adheres to our policy

Data sharing not applicable to this article as no datasets were generated or analysed during the current study.

Human research participants

Policy information about studies involving human research participants and Sex and Gender in Research.

Reporting on sex and gender	Not applicable.
Population characteristics	Not applicable.
Recruitment	Not applicable.
Ethics oversight	Not applicable.

Note that full information on the approval of the study protocol must also be provided in the manuscript.

Field-specific reporting

Please select the one below that is the best fit for your research. If you are not sure, read the appropriate sections before making your selection.

☒ Life sciences ☐ Behavioural & social sciences ☐ Ecological, evolutionary & environmental sciences

For a reference copy of the document with all sections, see nature.com/documents/nr-reporting-summary-flat.pdf

Life sciences study design

All studies must disclose on these points even when the disclosure is negative.

Sample size	Power analysese were performed to determine sample size in each experiment, using a desired power of 0.8 and alpha level of 0.05.
Data exclusions	Data were excluded from analysis of immunohistochemical images if samples exhibited poor immunostaining quality, or if samples did not contain sections with clear graft tissue visible.
Replication	To verify reproducibility, two different experimental cohorts were generated. Immunohistochemical experiments were performed twice. Genotyping was performed three times. All attempts at replication were successful.
Randomization	Animals in the histological studies were randomly assigned to treatment group using a random number generator internet page. Animals (Fig. 6) in the behavioral study were assigned to treatment group based on BMS (locomotor) scores prior to treatment, in order to ensure that there were no statistically significant differences in pre-treatment locomotor performance between groups.
Blinding	All investigators were blinded to the treatment during animal experiments and data analysis.

Reporting for specific materials, systems and methods

We require information from authors about some types of materials, experimental systems and methods used in many studies. Here, indicate whether each material, system or method listed is relevant to your study. If you are not sure if a list item applies to your research, read the appropriate section before selecting a response.

Materials & experimental systems

n/a	Involved in the study
☐	☒ Antibodies
☒	☐ Eukaryotic cell lines
☒	☐ Palaeontology and archaeology
☐	☒ Animals and other organisms
☒	☐ Clinical data
☒	☐ Dual use research of concern

Methods

n/a	Involved in the study
☒	☐ ChIP-seq
☒	☐ Flow cytometry
☒	☐ MRI-based neuroimaging

Antibodies

Antibodies used	A complete list of all the primary and secondary antibodies used in this study is provided in Supplementary Table 1.
Validation	Each primary antibody was validated in mouse spinal cord tissue sections by using a secondary-only control condition (in which no primary antibody was applied, but the appropriate species of fluorescently-conjugated secondary antibody was used), and confirming

lack of fluorescent signal. Antibodies were also validated by ensuring that patterns of immunoreactivity in mouse spinal cord tissue are similar to previously published historical controls.

Animals and other research organisms

Policy information about studies involving animals; ARRIVE guidelines recommended for reporting animal research, and Sex and Gender in Research

Laboratory animals	A total of n=310 mice were used for this study, including n=198 8-10 week-old C57BL/6J mice (#000664, Jackson Laboratories; n=76 males and n=122 females), n=4 P21 male and female C57BL/6 mice, n=30 3-8 month-old male homozygous GFP mice [C57BL/6-Tg(CAG-EGFP)131Osb/LeySopJ; #006567, Jackson Laboratories], n=18 3-8 month-old male homozygous Syn1-cre mice [B6.Cg-Tg(Syn1-cre)671Jxm/J, #003966, Jackson Laboratories], n=24 8-10 week-old homozygous female RϕGT mice [B6;129P2-Gt(ROSA)26Sortm1(CAG-RABVgp4,-TVA)Arenk/J, #024708, Jackson Laboratories], n=18 3-8 month-old homozygous male Chx10-cre mice [Tg(Vsx2-cre)TC9Gsat/Mmucd, MMMRRC 36672], and n=18 8-10 week-old homozygous female Ai14 mice [B6.Cg-Gt(ROSA)26Sortm14(CAG-tdTomato)Hze/J, #007914, Jackson Laboratories]. A total of n=12 F344 rats (F344/NHsd, Envigo) and n=6 homozygous GFP F344 rats [F344-Tg(UBC-#GFP)F455Rrrc, Rat Resource and Research Center #307] were used for this study. Animals had free access to food and water throughout the study and were group-housed in ventilated cages on a 12-hour light / 12-hour dark cycle (light cycle = 6:00 am – 6:00 pm), with ambient temperature between 20-23°C and 30-70% humidity. During the study, 5 animals died during surgery or post-operative recovery.
Wild animals	This study did not utilize wild-caught animals.
Reporting on sex	For the host animals that received spinal cord injury + transplantation (Data in Fig. 2-5), we used male mice. For the host animals that received spinal cord injury + transplantation for the behavior study (Data in Fig. 6), female mice were used.
Field-collected samples	The study did not utilize samples collected from the field.
Ethics oversight	All animal experiments were approved by the Texas A&M University Institutional Animal Care and Use Committee.

Note that full information on the approval of the study protocol must also be provided in the manuscript.
