# Supplementary material for: Developmental stage of transplanted neural progenitor cells influences anatomical and functional outcomes after spinal cord injury in mice
Source: Commun Biol. 2023 May 19;6:544. doi: 10.1038/s42003-023-04893-0 (PMC10199026; doi:10.1038/s42003-023-04893-0)
Supplement: Supplementary file 1 — Dulin_Peer Review File [file 42003_2023_4893_MOESM1_ESM.pdf]

nature portfolio

Peer Review File

Developmental stage of transplanted neural progenitor cells influences anatomical and functional outcomes after spinal cord injury in mice

Open Access This file is licensed under a Creative Commons Attribution 4.0 International License, which permits use, sharing, adaptation, distribution and reproduction in any medium or format, as long as you give appropriate credit to the original author(s) and the source, provide a link to the Creative Commons license, and indicate if changes were made. In the cases where the authors are anonymous, such as is the case for the reports of anonymous peer reviewers, author attribution should be to 'Anonymous Referee' followed by a clear attribution to the source work. The images or other third party material in this file are included in the article's Creative Commons license, unless indicated otherwise in a credit line to the material. If material is not included in the article's Creative Commons license and your intended use is not permitted by statutory regulation or exceeds the permitted use, you will need to obtain permission directly from the copyright holder. To view a copy of this license, visit http://creativecommons.org/licenses/by/4.0/.

Parts of this Peer Review File have been redacted as indicated to maintain the confidentiality of unpublished data.

Reviewers' comments:

Reviewer #1 (Remarks to the Author):

The manuscript, "Developmental stage of transplanted neural progenitor cells influences anatomical and functional outcomes after spinal cord injury", is about the specific age of embryonic rodent NPCs influencing graft cellular composition, graft axon outgrowth, host axon regeneration, and sensory behavioral outcomes following transplantation into the lesioned spinal cord. This topic is very important to trace cellular identity in transplantation research. In this study, developmentally-restricted spinal cord NPCs, dissected from E11.5-E13.5 mouse embryos, were transplanted into sites of adult mouse SCI. Immunostaining and sensorimotor behavioral tests were used to evaluated cellular composition and axon growth. The results demonstrated significant difference between earlier- and later- stage grafts: earlier-stage grafts exhibited greater axon outgrowth, enrichment for ventral spinal cord interneurons and Group-Z spinal interneurons, and enhanced host 5-HT+ axon regeneration, whereas Later-stage grafts were enriched for late born dorsal horn interneuronal subtypes and Group-N spinal interneurons, supported more extensive host CGRP+ axon ingrowth, and exacerbated thermal hypersensitivity. The study has implications for experimental SCI/transplantation research. Although they did not perform corticospinal tracing in this study, it was pointed out the interest to see how graft cellular composition influences regeneration of this in the future. The experiments were designed very well and the manuscript were well written. The methods were described clearly and reported results were sound and interpreted pertinently. The reviewer really enjoyed when reading through the manuscript. Thus, this reviewer recommends publishing this high-quality manuscript with strong enthusiasms after a few minor concerns are addressed.

1. In the methods, SCI surgeries section, because mouse and rat embryonic cells were used in the study, it would be better to use "mice" instead of "animals" to pass clearer information.

2. Two injury models, dorsal column lesion and contusion, were included. In the results, this may be stated in every section for clarification.

3. In line 661, change "GFP+" to "GFP+(supercript)".

Reviewer #2 (Remarks to the Author):

In this manuscript, the authors examined whether transplantation of different stages of neural progenitor cells (NPCs) affects graft cellular components, graft derived axonal outgrowth, host axon regeneration and behaviors after spinal cord injury (SCI). They mainly used mouse thoracic contusive SCI models and transplanted embryonic spinal cord derived NPCs from embryonic day 11.5, 12.5, and 13.5 embryos. They found that earlier-stage E11.5 grafts contained more ventral spinal cord interneurons and Group-Z spinal interneurons, had more axonal extension and enhanced host 5-HT+ axon regeneration. Later-stage E13.5 grafts had more late born dorsal horn interneuronal subtypes and Group-N spinal interneurons, supported more extensive host CGRP+ axon ingrowth, and exacerbated thermal hypersensitivity.

This is a comprehensive study to systematically characterize the stages of NPCs that affect the graft outcomes. The identification of early-stage NPCs that had more ventral interneurons, more axonal extension, and more host axonal regeneration is significant and provides practical guidance for NPC transplantation studies in rodent models of SCI.

A few of minor questions:
1) Did early-stage NPCs survived better to completely fill the lesion site than the late stage NPCs?

2) Can authors show examples of graft-derived axonal extension images since the authors only provided the quantification data in Fig. 2I?
3) The pattern of staining in Fig. 1 indicates that mouse NPCs were cultured as neurospheres where it is hard to count individual cells/nucleus. Why not cultured them as adherent culture as a single layer of cells so it is easy to count labeled cells?
4) Line 717, How the axon outgrowth quantification was sampled and calculated?

Reviewer #3 (Remarks to the Author):

This study highlights the inconsistencies present in literature that describe an ideal time point in which primary derived neural progenitor cells (NPCs) should be isolated from embryonic murine tissue. The authors investigate timepoints E11.5-E13.5, which exhibit different migratory patterns, differentiation profiles, and effects on functional recovery following post-spinal cord injury (SCI) transplantation. This is a very interesting study that implies different grafting dynamics between transplanted and endogenous tissue, possibly due to neuronal subtype specification. However, this description is correlational (i.e., we see different neuronal subtypes in grafts if we take the same dissected tissue from different developmental time points) and would largely benefit from proof-of-concept (i.e., are these neuronal subtypes necessary for producing these differential functional outcomes and grafting dynamics?).
This was an exciting read and I strongly recommend accepting this work, following the corrections below.
Critiques of the work:
• NPCs derived from different embryonic stages likely have different proliferative capacities. As the embryonic spinal cord reaches peak neurogenesis by E12.5, it stands true that there is likely a greater proliferative capacity prior to this developmental stage. Based on Figure 2i, earlier stage grafts don't necessarily mean enhanced axonal outgrowth, but likely imply greater survival, as there are more GFP+ axons found at either rostral/caudal position beyond the lesion site.

• *As primary cells are grafted from a spinal cord homogenate, there is likely a combination of NPCs and immature/mature cell types present in the cell suspension. Are the differences in neuronal subtype specification due to the differential potency of NPCs present in the cell suspension, or simply the presence of different mature cell types at developmental time points?

o This terminology should be clarified throughout the manuscript; as NPCs are likely the most abundant cell time present in the suspension (and the most likely to survive and proliferate), however, it likely contains other maturing cell types.

• Pertaining to Figure 1
o Inconsistencies throughout the manuscript regarding what type of SCI is performed (thoracic or cervical; re: figure 2 and results descriptions of SCI)
o A better descriptor is required for the NPCs grown in figure 1. Are these neurospheres, or cells grown in an adherent culture system?
o There are several differences between cell fates isolated from different embryonic stages (Chx10, FoxP2, Sox2) which requires more discussion and referencing (re: * point above)
o Do you see a difference between the neuronal subtype identity of cells between in vitro and in vivo SCI? A discussion/comparison here should be made and why differences here exist.

• Methodology
o Very little detail/description regarding cell transplantation (i.e., rostral/caudal to the contusion, how many transplant sites, what volume, etc.)

• No functional benefits (motor) of any NPC graft to promote recovery after SCI

o A more thorough discussion here is required. This is likely due to the level of injury severity (contusion was likely not heavy enough) or there may have not been enough cell transplanted, in the proper location, etc. (re: methodology point)
o H&E stain or assessment of lesion cavity size can address the severity of injury
• Staining quantification, overlay with dapi to ensure particles are not artifacts
• Innervation vs axon regeneration in figure 5; this phrasing is a bit misleading as staining only suggests one term

We thank the reviewers for their time and effort in reviewing our manuscript. We appreciate the strong positive feedback about our study and all of the productive comments that were provided. We have carefully considered all of the reviewers' comments and integrated the suggested changes into the revised version of the manuscript. Please see below for a point-by-point response to reviewers' comments (our responses are in blue). Note that the revised manuscript has all changes highlighted.

Reviewer #1:

The manuscript, "Developmental stage of transplanted neural progenitor cells influences anatomical and functional outcomes after spinal cord injury", is about the specific age of embryonic rodent NPCs influencing graft cellular composition, graft axon outgrowth, host axon regeneration, and sensory behavioral outcomes following transplantation into the lesioned spinal cord. This topic is very important to trace cellular identity in transplantation research. In this study, developmentally-restricted spinal cord NPCs, dissected from E11.5-E13.5 mouse embryos, were transplanted into sites of adult mouse SCI. Immunostaining and sensorimotor behavioral tests were used to evaluated cellular composition and axon growth. The results demonstrated significant difference between earlier- and later- stage grafts: earlier-stage grafts exhibited greater axon outgrowth, enrichment for ventral spinal cord interneurons and Group-Z spinal interneurons, and enhanced host 5-HT+ axon regeneration, whereas Later-stage grafts were enriched for late born dorsal horn interneuronal subtypes and Group-N spinal interneurons, supported more extensive host CGRP+ axon ingrowth, and exacerbated thermal hypersensitivity. The study has implications for experimental SCI/transplantation research. Although they did not perform corticospinal tracing in this study, it was pointed out the interest to see how graft cellular composition influences regeneration of this in the future. The experiments were designed very well and the manuscript were well written. The methods were described clearly and reported results were sound and interpreted pertinently. The reviewer really enjoyed when reading through the manuscript. Thus, this reviewer recommends publishing this high-quality manuscript with strong enthusiasms after a few minor concerns are addressed.

1. In the methods, SCI surgeries section, because mouse and rat embryonic cells were used in the study, it would be better to use "mice" instead of "animals" to pass clearer information.
We thank the reviewer for the suggestion to clarify the species of animals used in all experiments. In all applicable instances within the Methods and the Results sections, we have now changed "animals" to "mice" or "rats". In fact, due to this comment we realized that we had inadvertently left out the methods for the rat SCI and transplantation surgeries, so this section has now been added to the Methods.

2. Two injury models, dorsal column lesion and contusion, were included. In the results, this may be stated in every section for clarification.
We thank the reviewer for this comment, and we have now clarified the injury model in the sections corresponding to each figure in the Results.

3. In line 661, change “GFP+” to “GFP+(supercript)”.
Done.

Reviewer #2:

In this manuscript, the authors examined whether transplantation of different stages of neural progenitor cells (NPCs) affects graft cellular components, graft derived axonal outgrowth, host axon regeneration and behaviors after spinal cord injury (SCI). They mainly used mouse thoracic contusive SCI models and transplanted embryonic spinal cord derived NPCs from embryonic day 11.5, 12.5, and 13.5 embryos. They found that earlier-stage E11.5 grafts contained more ventral spinal cord interneurons and Group-Z spinal interneurons, had more axonal extension and enhanced host 5-HT+ axon regeneration. Later-stage E13.5 grafts had more late born dorsal horn interneuronal subtypes and Group-N spinal interneurons, supported more extensive host CGRP+ axon ingrowth, and exacerbated thermal hypersensitivity.

This is a comprehensive study to systematically characterize the stages of NPCs that affect the graft outcomes. The identification of early-stage NPCs that had more ventral interneurons, more axonal extension, and more host axonal regeneration is significant and provides practical guidance for NPC transplantation studies in rodent models of SCI.

A few of minor questions:
1) Did early-stage NPCs survived better to completely fill the lesion site than the late stage NPCs?
Actually, we observed equivalent survival and filling of grafts in all groups. We never observed any “holes” in grafts. We noted this in the manuscript Results section, in the paragraph that discusses Figure 2: “*At four weeks post-transplantation, all grafts exhibited strong GFP fluorescence and complete filling of the lesion site*”.

As a side note that I hope will be helpful to reviewers and readers, I believe that our observation that all grafts show complete filling of the lesion site and good survival is because we graft high concentrations of cells (~$4x10^5$ cells/μL). This gives the NPCs a high chance to survive and proliferate, even if large numbers of cells die after grafting. In my experience, I have only ever had poorly-surviving grafts with “holes” if I grafted lower concentrations of cells (e.g., 2 $x10^5$ cells/μL).

2) Can authors show examples of graft-derived axonal extension images since the authors only provided the quantification data in Fig. 2I?
Yes, we have now provided some example images in the new **Supplementary Figure 2**. (The other two Supplementary Figures have now been re-numbered.)

3) The pattern of staining in Fig. 1 indicates that mouse NPCs were cultured as neurospheres where it is hard to count individual cells/nucleus. Why not cultured them as adherent culture as a single layer of cells so it is easy to count labeled cells?

In fact, we did culture cells as adherent cultures in a single layer. The appearance of 'neurospheres' occurs because some small clusters of cells seem to proliferate more than others. You can see this phenomenon in a previous publication (Dulin et al, *Nat Comms*, 2018; Figure 1k). I am including an example image of DAPI^{+} cells in culture below so the reviewers can appreciate the appearance of the "cell clusters" present in adherent cultures. Note that the brightness and contrast of this image has been increased so that all cells can be seen. We used a microscope with an extended depth of Z-focus function to make sure that we counted all cells accurately.

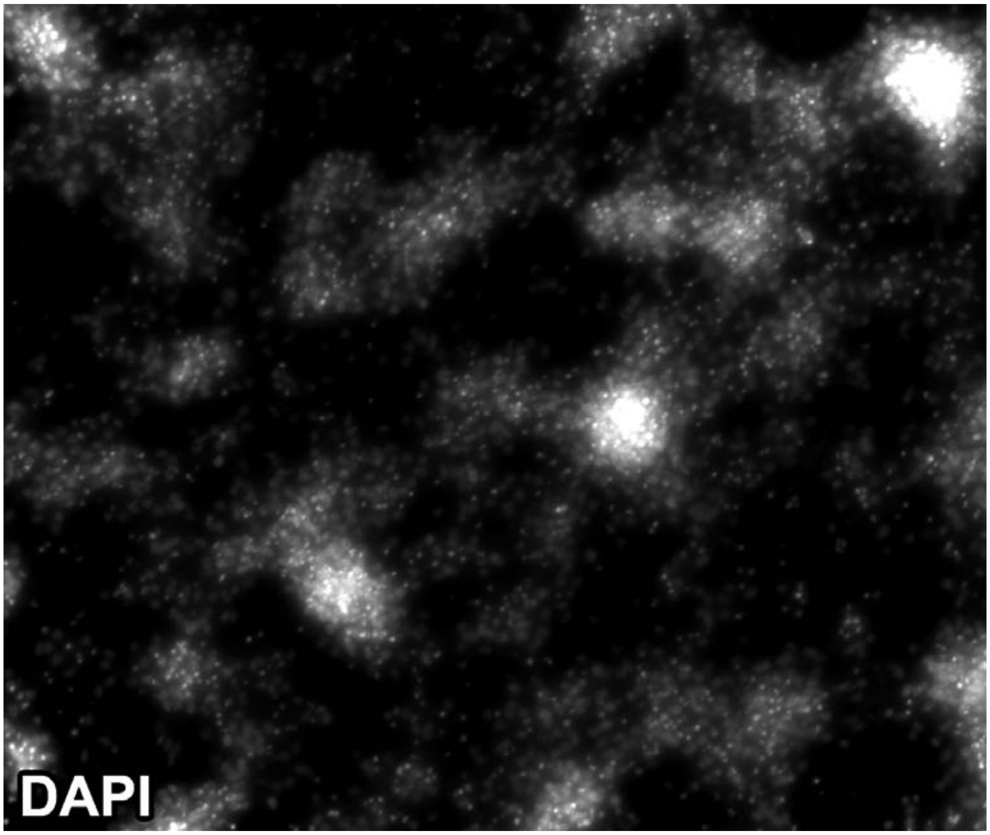

4) Line 717, How the axon outgrowth quantification was sampled and calculated?

We have now provided more detail in the Methods section:

"<u>Axon outgrowth quantification</u>: A 1-in-6 series of tissue was used to calculate axon outgrowth. GFP fluorescence was overexposed so that fine GFP^{+} processes were visible at sites distant from the main body of the graft. Graft ROIs were translated in 250-μm increments for 2 mm in rostral and caudal directions throughout the host spinal cord. At each increment, the total number of GFP^{+} axons crossing the leading edge of the ROI was manually counted. The total number of axons in the 1-in-6 tissue series was multiplied by 6 to obtain the extrapolated axon outgrowth for the whole graft, and then this number was divided by the graft volume [(graft area in each ROI) x 6 x section thickness]. Data are represented as the total graft axon outgrowth, normalized to graft volume."

Reviewer #3:

This study highlights the inconsistencies present in literature that describe an ideal time point in which primary derived neural progenitor cells (NPCs) should be isolated from embryonic murine tissue. The authors investigate timepoints E11.5-E13.5, which exhibit different migratory patterns, differentiation profiles, and effects on functional recovery following post-spinal cord injury (SCI) transplantation. This is a very interesting study that implies different grafting dynamics between transplanted and endogenous tissue, possibly due to neuronal subtype specification. However, this description is correlational (i.e., we see different neuronal subtypes in grafts if we take the same dissected tissue from different developmental time points) and would largely benefit from proof-of-concept (i.e., are these neuronal subtypes necessary for producing these differential functional outcomes and grafting dynamics?). This was an exciting read and I strongly recommend accepting this work, following the corrections below.
We thank the reviewer for this positive feedback. We agree the outcomes are correlative and that proof of concept studies are needed to implicate specific cell types in functional outcomes. Indeed, we are currently wrapping up a separate study in which we examine the functional contributions of specific graft neuronal subtypes to muscle activation and locomotor functional outcomes. Ongoing work in the lab is also examining how enriching grafts for these cell types affect functional outcomes.

Critiques of the work:
NPCs derived from different embryonic stages likely have different proliferative capacities. As the embryonic spinal cord reaches peak neurogenesis by E12.5, it stands true that there is likely a greater proliferative capacity prior to this developmental stage. Based on Figure 2i, earlier stage grafts don’t necessarily mean enhanced axonal outgrowth, but likely imply greater survival, as there are more GFP+ axons found at either rostral/caudal position beyond the lesion site.
This is a great point. We agree that NPCs derived from different developmental stages are undergoing different degrees of proliferation after culturing and grafting. All grafts had about the same neuronal density, except E11.5 which were significantly less dense than E12.5. However, these grafts had more axon outgrowth (normalized to graft size). This suggests that the greater axon outgrowth is not due to greater survival, but due to <u>greater axon outgrowth per neuron</u> compared with the other groups. One potential reason for this could be that E11.5 grafts are more enriched for neuronal subtypes that normal project longer distances (e.g. propriospinals). We have now noted this in the Discussion section:

“*We analyzed abundances of Group-N and Group-Z cells in grafts; these are recently-described classifications of spinal cord neurons that go beyond the cardinal classes of neurons, dividing them into their “motor-sensory, local-long range, and excitatory-inhibitory features”*[83]*. Consistent with the findings of that study, here we observed greater abundance of the late-born Group-N neurons in later-stage E13.5 grafts, and greater abundances of the early-born Group-Z neurons in the earlier-stage E11.5 grafts. One hallmark of Group-Z neurons described by Osseward et al. is that they are projection neurons with long-distance axon projects to the thalamus, cerebellum, and brainstem. Here, we observed greatest axon outgrowth in E11.5 grafts, despite*

these grafts having a lower neuronal density than E12.5 grafts, suggesting that there is greater axon outgrowth per neuron in the E11.5 group. We speculate that this could potentially be attributed to the enrichment of these grafts for long-distance projection neurons, such as Group-Z projection neurons. Further work is needed to explore the subtypes of neurons that send long-distance projections in grafts, as these may be critical for relaying motor signals to the caudal spinal cord. We also observed differential regeneration of 5-HT^{+} and CGRP^{+} host axons into grafts. Given the importance of these axons in motor and sensory function in the intact spinal cord, respectively, this suggests that earlier-stage grafts may be better suited to studies of graft integration with motor circuits, and later-stage grafts might be better suited to studies of graft integration with sensory or pain circuits. We did not perform corticospinal tracing in this study, but it would be interesting to how graft cellular composition influences regeneration of this and other functionally important axon projections into grafts after SCI."

*As primary cells are grafted from a spinal cord homogenate, there is likely a combination of NPCs and immature/mature cell types present in the cell suspension. Are the differences in neuronal subtype specification due to the differential potency of NPCs present in the cell suspension, or simply the presence of different mature cell types at developmental time points? This terminology should be clarified throughout the manuscript; as NPCs are likely the most abundant cell time present in the suspension (and the most likely to survive and proliferate), however, it likely contains other maturing cell types.
This is also a great point. We suggest in the manuscript that the differences in subtype abundances is due to the differential potency of the NPCs present in the cell suspension, but we agree there are other cells (postmitotic neurons) present in the suspension that could be contributing to graft diversity. We have now added sentences discussing this in the Figure 1 section of the Results:

"*Additionally, it is important to note that due to the ages of embryonic development from which these cells were obtained, the initial cell suspensions likely contain a mixture of NPCs and postmitotic neurons[26]. This is supported by the observation that not all cells express the NPC marker Sox2 after 24 h in culture (**Fig. 1c**). However, for the purposes of this study we will refer to the cell preparations as "NPCs", because the progenitors are the cells that are most capable of survival and proliferation after transplantation, and therefore these cells make up the majority of the mature graft.*"

Pertaining to Figure 1
o Inconsistencies throughout the manuscript regarding what type of SCI is performed (thoracic or cervical; re: figure 2 and results descriptions of SCI)
Thoracic SCI was only performed in Figure 6; the rest were cervical SCI. Please see response to Reviewer #1 above; we have now clarified the injury model in the sections corresponding to each figure in the Results.

o A better descriptor is required for the NPCs grown in figure 1. Are these neurospheres, or cells grown in an adherent culture system?
Please see response to Reviewer #2. We cultured cells as adherent cultures in a single layer. The appearance of neurospheres occurs because some small clusters of cells seem to proliferate more than others. You can see this phenomenon in a previous publication (Dulin et al, *Nat Comms*, 2018; Figure 1k), and an example image is included above.

o There are several differences between cell fates isolated from different embryonic stages (Chx10, FoxP2, Sox2) which requires more discussion and referencing (re: * point above)
o Do you see a difference between the neuronal subtype identity of cells between in vitro and in vivo SCI? A discussion/comparison here should be made and why differences here exist.
We have now added a paragraph to the Discussion expounding on both of these points:

"*In grafts placed into sites of SCI in vivo, we observed significant differences in the abundance of multiple neuronal subtypes among grafts of different developmental NPC stages. For example, ventral spinal cord V1 and V2a interneuron populations were enriched in earlier-stage grafts, and dorsal horn neuronal populations (dILA, dILB, laminae I-III calbindin$^+$ clusters) were enriched in later-stage grafts. These observations closely reflect the differences in subtype abundance among E11.5, E12.5, and E13.5 NPCs that we observed after 72 h culture in vitro. Upon analyzing the distribution of distinct NPC lineages in vitro, we found that ventral progenitors (Pax6$^+$ and Nkx2.2$^+$ progenitors) were enriched in earlier-stage cells. Collectively, these findings corroborate what is known about the abundances of distinct dorsal and ventral spinal cord NPC populations during the neurogenic period; generally, that ventral progenitors are most proliferative at E10-E11 and dorsal progenitors are most proliferative at later stages[26]. Together, our data provide evidence that NPC cell fates are determined intrinsically during spinal cord development, prior to isolation and culture or transplantation of these cells, and that these fates are retained regardless of whether they are cultured in vitro or transplanted in vivo. This is unsurprising, given earlier work in the field of fetal spinal cord transplantation showing that fetal grafts placed into heterotopic environments (e.g., the brain, the anterior chamber of the eye) retained morphological characteristics of the adult spinal cord[20,21,24,91].*"

Methodology
o Very little detail/description regarding cell transplantation (i.e., rostral/caudal to the contusion, how many transplant sites, what volume, etc.)
We have provided detailed information about the transplantation in the Methods section under the subheading "NPC transplantation" (starting at line 578 in the manuscript), including exact anatomical coordinates of injection, how many transplant sites, and how much volume was used for each SCI model.

No functional benefits (motor) of any NPC graft to promote recovery after SCI
o A more thorough discussion here is required. This is likely due to the level of injury severity (contusion was likely not heavy enough) or there may have not been enough cell transplanted, in the proper location, etc. (re: methodology point)

We agree with the reviewer that these are valid concerns. To address this, we quantified the volume and neuronal density of the grafts placed into the contusion sites to determine whether there was poor graft survival or not enough cells. This data is now included in **Figure 6c-d**. We did not find any significant differences in these outcomes between treatment groups, and all of the subjects for which locomotor scores are reported showed surviving grafts densely populated with neurons. Hence, the amount of cells transplanted or the degree of graft survival is not likely to be the reason why we failed to observe locomotor recovery. To address the question of lesion severity: In the manuscript, we chose to represent the scores as “fold change from pre-treatment scores” but I am including here for review purposes the raw BMS data for each group (please keep in mind treatment was administered at 14 days post-SCI). From this data, we hope the reviewers can appreciate that this is a moderate-to-severe lesion, with animals recovering to a plateau of 2-3. In fact, only one animal was consistently stepping in this entire cohort. However, there are no significant differences between treatment groups (Time x treatment effect: P = 0.7609 by two-way repeated measures ANOVA).

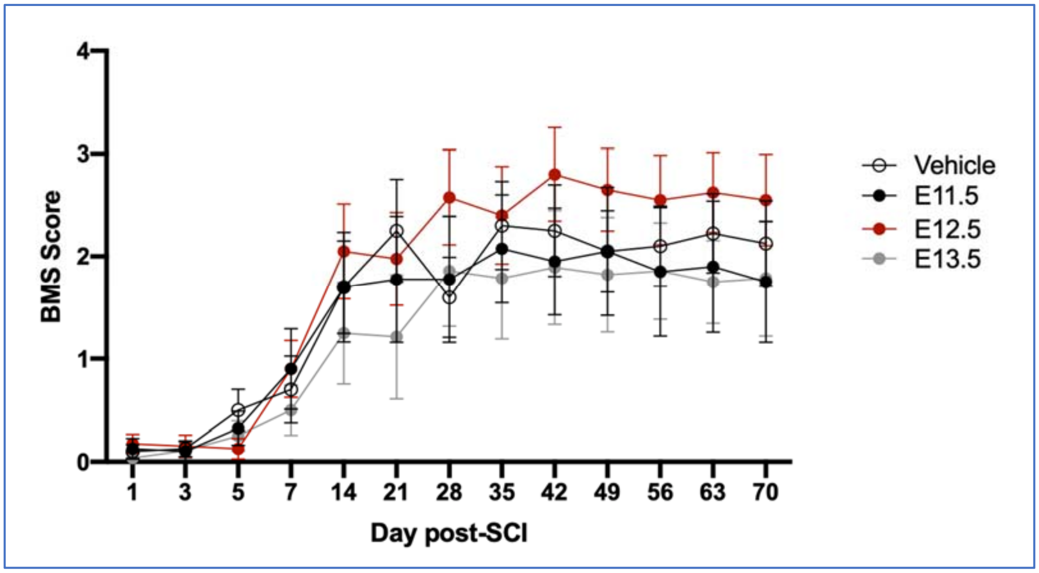

We feel that the most likely explanation for the failure of NPC grafts to promote recovery after SCI in this lesion/grafting model is because graft-derived neurons aren’t synaptically & functionally integrating with locomotor circuitry to the degree that is required to elicit behavioral improvement. We have transsynaptic tracing data to suggest this is the case (unpublished data for another study). In addition, a graduate student in my lab ran a large cohort of mice utilizing a T12 moderate-to-severe contusion SCI (N=17 SCI only, N=29 SCI + E12.5 NPC graft). We did not observe any significant Time x Treatment effect (P=0.638). Please see data below.

[figure redacted]

o H&E stain or assessment of lesion cavity size can address the severity of injury
Thank you for the suggestion. Because these are mice, it is difficult to assess the lesion cavity size since there is no cavity that forms following contusion in mice.

• Staining quantification, overlay with dapi to ensure particles are not artifacts
Yes, we did this for all quantifications. We have now added the following sentence to the Methods section: "*Cell type-specific markers were always overlaid with the DAPI channel during analysis to ensure that any staining artifacts were not included in quantification.*"

• Innervation vs axon regeneration in figure 5; this phrasing is a bit misleading as staining only suggests one term
Thank you for the suggestion. We have now made the wording more consistent to refer to "regeneration" rather than "innervation".

REVIEWERS' COMMENTS:

Reviewer #2 (Remarks to the Author):

The authors addressed reviewers' concern and the manuscript is in a good shape for publication.
